# Strain-specific outcomes of cytosine-base editing in *Streptomyces*

**DOI:** 10.1128/jb.00241-26

**Published:** 2026-09-18

**Authors:** Maxime M. Boneza, Thomas Keller, Josephine Mostek, Michael J. Smanski

**Affiliations:** 1 Department of Biochemistry, Molecular Biology, and Biophysics, University of Minnesota 200766, Minneapolis, Minnesota, USA; 2 Biotechnology Institute, University of Minnesota, Saint Paul, Minnesota, USA; National Institutes of Health, Bethesda, Maryland, USA

**Keywords:** *Streptomyces*, base editors, DNA repair, transversion mutations

## Abstract

The clustered regularly interspaced short palindromic repeats (CRISPR)-Cas system has facilitated gene editing of different organisms. Specifically, programmable base editing enables stable conversion of a single nucleotide to another nucleotide without causing DNA double-strand breaks. Cas9-derived base editors, including adenine and cytidine base editors, catalyze the formation of transition mutations with high efficiency. The third-generation cytidine base editors comprise a nickase-Cas9 fused to a cytidine deaminase and uracil DNA glycosylase inhibitor to enable the transition from C:G to T:A. We observed that in certain *Streptomyces* spp., this cytidine base editor produced C:G to G:C transversions at a surprisingly high rate of 44, while in other strains, it yielded the expected C:G to T:A transition mutations. There was also a notable timing difference for base editing between distinct strains. Bioinformatics analysis revealed differences in DNA mismatch repair and nucleotide excision repair pathways, including the presence of *uvrD* helicase gene only in the C:G to G:C transversion strain. Expression of *uvrD* in the C:G to T:A transition strain led to higher rates of C:G to G:C transversions. These discoveries will aid in the development of efficient C:G to G:C base editors.

**IMPORTANCE:**

Base editors are useful tools that allow genetic engineers to precisely change single bases in an organism's genome. Most base editors catalyze transition mutations (e.g., A-to-G or C to-T). Transversion mutations, which convert a purine base to a pyrimidine base, have been described but operate at much lower efficiency. Here, we describe a surprising discovery that a transition base editor produced high-efficiency transversion mutations in a species of *Streptomyces*. This information was used to engineer a high-efficiency transversion base editor.

## INTRODUCTION

**Fig 1:**
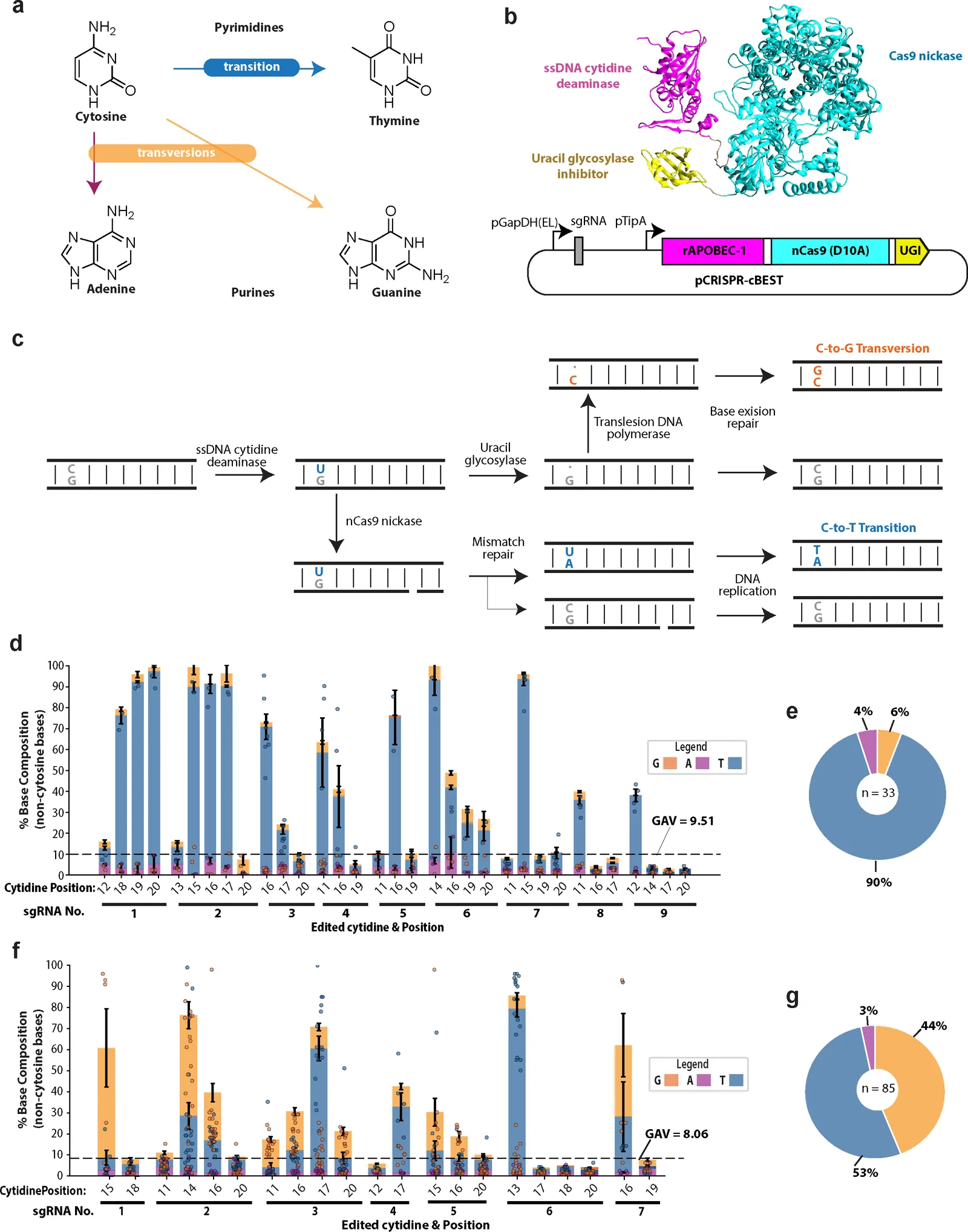
Differential cytosine base editing in two strains of *Streptomyces* species. (**a**) Types of DNA base conversions. CBEs induce C -> T transitions, while ABEs (not discussed in this work) induce A -> G DNA base transitions. (**b**) BE3 complex structure as predicted by AlphaFold. The domains are shown as follows: APOBEC-1 (magenta), nCas9 (cyan), and uracil DNA glycosylase inhibitor (yellow). The arrangement of these genes on the plasmid is shown. (**c**) Mechanism of cytosine base editing. nCas9 forms the R-loop and creates a DNA single-strand break. Formation of the R-loop enables cytidine deaminase to act on ssDNA, deaminating cytosine to uridines, which pair with DNA base G. Subsequently, uracil DNA glycosylase inhibitor prevents reversion to cytosine, leading to DNA mismatch repair and a round of DNA replication, creating U:A base pairing and further repair to T:A. Alternatively, through unknown conditions and mechanisms, uracil DNA glycosylase creates an abasic site. Translesion DNA polymerases and base excision repair resolve the abasic site to G, leading to C:G base pairing. (**d**) BE3 base editing results across nine different target sites in *Streptomyces* Strain A are shown. The cytidine position, PAM-distal in the editing window, is shown directly under each bar for each target site. BE3 products are transitions of pyrimidines where the C:G DNA base pair is converted to the T:A DNA base pair, as expected. (**e**) Overall base editing in Strain A. The majority of BE3 products are transitions from C:G to T:A at 89.6%,of C:G to T:A products not including positions where editing is below the dashed horizontal line. (**f**) BE3 base editing across six sites in *Streptomyces* Strain B. The target site is shown, and the edited cytidine is displayed directly under each bar. This *Streptomyces* produces both pyrimidine transitions and pyrimidine to purine transversion base editing. (**g**) The overall editing in *Streptomyces* Strain B shows a 52.8 pyrimidine transition (C:G to T:A) and a 44.0 pyrimidine to purine transversion (C:G to G:C). This overall product in *Streptomyces* Strain B is an anomaly. For (**d**) and (**f**), the dashed lines labeled GAV are a measure of the average background noise across the sequenced region (Methods). The error bar is the standard error of the mean. The raw data points are shown for each position within the bar graph. For panels **e** and **g**, the pie-chart data are weighted to account for dissimilar number of biological replicates.

Cas9-derived base editors (BEs) allow genetic engineers to make targeted single-base changes to a DNA sequence (1–7). Because BEs avoid introducing double-strand breaks, they circumvent the error-prone non-homologous end-joining DNA repair pathway (8–10). The most commonly used BEs drive the formation of transition mutations: cytosine (C) to thymine (T) or adenine (A) to guanine (G) CBEs or ABEs (Fig. 1a). High-efficiency CBEs are multidomain engineered proteins that comprise: (i) a Cas9-nickase (nCas9), which contains an active site mutation that abrogates cleavage of the edited strand; (ii) a cytidine deaminase (e.g., APOBEC-1); and (iii) a uracil DNA glycosylase inhibitor (UGI) (Fig. 1b).

Achieving high-efficiency C to T mutations requires guiding the endogenous DNA repair pathways toward the desired outcome. A single-guide RNA (sgRNA) directs the APOBEC-1-nCas9-UGI protein complex to the target site, where base-pairing between the sgRNA and complementary target sequence exposes an R-loop of single-stranded DNA. APOBEC-1 deaminates one or multiple C bases to form uracil (U) within an 8-base editing window of the sgRNA on the unpaired DNA strand in the R-loop, while the nCas9 breaks a phosphodiester bond on the opposite strand. UGI prevents excision of the U from the U:G mismatch via base excision repair (1). Instead, cells interpret the nicked strand of DNA as the lesion-containing strand, and single-strand break repair (SSBR) or mismatch repair (MMR) replaces the G in the U:G mismatch with an A. This U:A base pair is converted to a T:A mutation during the next round of DNA replication (Fig. 1c). Several generations of base-editor designs use slight modifications of this process (5, 11–13).

Transversion mutations, including C to G, have been challenging to achieve with high efficiency. Several groups have sought to engineer C to G BEs (CGBEs) in mammalian cells (4, 11, 14). These transversion mutations are achieved by replacing the UGI domain with an *E. coli* uracil DNA N-glycosylase (eUNG) (14), with DNA repair protein (rXRCC1) (11), or with a UNG ortholog from *Mycobacterium smegmatis* (UdgX) (4). The efficiency of these transversion BEs varies greatly depending on the target site but tends to be moderate. For example, average (and standard deviation) editing yields for all reported C to G conversions are 15% ± 18% for CGBE1 from Kurt et al. (14), 22% ± 8% for the rA1 variants from Chen et al. (11), and 16% ± 14% for the 12 best-performing CGBEs from Koblan et al. (4). We note that Koblan et al. developed a machine-learning model that allowed for bioinformatic-guided selection of an optimal CGBE design for a particular sequence. The average editing yield reported in the previous sentence is for their 12 best-performing CGBEs across non-prioritized targets. High-efficiency CGBEs would have potential therapeutic applications in correcting 11 (3,525 SNVs) of the known disease-causing single-nucleotide polymorphisms in humans (3).

Here, we describe an unexpected finding when using the third-generation cytidine base editors (BE3) (Fig. 1c) to modify the genomes of non-model bacteria within the genus *Streptomyces. Streptomyces* spp. are known as prolific producers of clinically relevant specialized metabolites. They have several noteworthy genetic traits, including large genomes, linear chromosomes, and high guanine-cytosine (GC) content (70–75%). CBEs that are codon-optimized for use in *Streptomyces* have been previously described (15–17) and used by many groups (15, 18). When using these tools to engineer the genome of *Streptomyces* spp. isolated from disease-suppressive soils (19), we found the BE outcomes to be highly divergent between different strains (see below). We characterize the rate of transversion mutation across several target sequences and show that it is a robust and reproducible finding. Bioinformatic comparison of the gene content in the two *Streptomyces* strains revealed divergence in DNA mismatch and nucleotide excision repair mechanisms that may be involved in the unique editing outcomes. Lastly, supplementation with DNA repair enzymes implicated the UvrD helicase as a major contributor to C:G to G:C base editing outcomes in *Streptomyces* Strain B.

## MATERIALS AND METHODS

### Strains, media, and chemicals

*Escherichia coli* NEB Stable, DH5α, or 10Beta were used for routine cloning depending on plasmid size. *Escherichia coli* ET12567/pUZ8002 was used for intergenic conjugation. *Streptomyces* strains A (Joint Genome Institute taxon ID: 2758568662) and B also known as *Streptomyces* sp. 3211-3 (19) (Joint Genome Institute taxon ID: 2863083887) were obtained from the Kinkel Lab *Streptomyces* strain collection at the University of Minnesota. Luria-Bertani (LB) medium (10 g/L tryptone, 5 g/L yeast extract, 10 g/L NaCl; Difco LB broth, Miller reference #: 244620) supplemented with necessary antibiotics was used for strain maintenance and plasmid amplification in *E. coli. Streptomyces* solid media for maintenance and spore preparation include Difco ISP Medium No. 4 (10 g/L soluble starch, 1 g/L dipotassium phosphate, 1 g/L magnesium sulfate USP, 1 g/L sodium chloride, 2 g/L ammonium sulfate, 2 g/L calcium carbonate, 20 g/L agar, 0.001 g/L ferrous sulfate, 0.001 g/L manganous chloride, 0.001 g/L zinc sulfate) reference #: 277210 and HIMEDIA ISP Medium 3 (20 g/L oatmeal, 1 mL trace salts solution, 18 g/L agar) reference #: M358-500G. Conjugation was performed on ILW4 (37 g/L Difco ISP Medium 4, 0.5 g/L yeast extract, 1 g/L tryptone) plates supplemented with 20 mM CaCl_2_. Exconjugants were grown on Difco ISP Medium No. 4 (37 g/L) media plates supplemented with appropriate antibiotics. Where necessary, apramycin (50 mg/mL), chloramphenicol (25 mg/mL), kanamycin (50 mg/mL), and nalidixic acid (50 mg/mL) antibiotics were utilized. CloneAmp, 2× GoTaq Green Master mix, NEB 2× Q5 polymerase, Promega T4 ligase, AarI restriction enzyme, paqC1 (alternatively, GeneArt AarI 2× mix), and NEB 2× HiFi Gibson assembly mix were used. Oligonucleotides used in this work were synthesized by Integrated DNA Technologies (IDT).

### Plasmid designs and assembly

The plasmid pCRISPR-cBEST was ordered from Addgene (catalog no. 125689). As designed, pCRISPR-cBEST allows insertion of a single-stranded guide RNA oligonucleotide via isothermal assembly cloning method. This assembly method results in fewer colonies post-sgRNA insertion. In addition, the lack of a reporter gene to differentiate the original plasmid from the newly assembled plasmid resulted in excessive screening of colonies that received the sgRNA. Therefore, to minimize screening and streamline sgRNA insertion, we modified the pCRISPR-cBEST plasmid to allow sgRNA insertion via Type IIS restriction cloning method. We also wanted to qualitatively screen colonies that received the sgRNA plasmid. As such, we created pMB520_cBEST with a lacZ cassette flanked by AarI restriction sites inserted upstream of the sgRNA scaffold for blue-white colony screening. The modified cBEST plasmid utilized this design. For sgRNA insertion, designed gRNAs were ordered from Integrated DNA Technologies (IDT) as complementary oligonucleotides: 5′-ACGC-Fwd-protospacer-3′ and 5′-AAAC-reverse complement protospacer-3′. Hydrated complementary oligonucleotides to 25 µmol/L were annealed (4.0 µL FWD and 4.0 µL REV and 2 µL H_2_O molecular bio grade) in a thermocycler using the following protocol: 5 min at 95°C and step down to 25°C for 1 h. The annealed oligo reaction was diluted to 1 in 250 µL H_2_O. Then, in a clean PCR tube, 1 µL of the diluted annealed oligo was mixed with 1 µL plasmid (50 ng/µL), 1 µL Promega T4 ligase buffer, 1 µL PaqC1 activator, 0.5 µL NEB PaqC1 enzyme, 0.5 µL Promega T4 DNA ligase, and molecular bio grade water to 10 µL. The reaction was run in a thermocycler with the following settings: 37°C for 15 min, 37°C for 2 min, and 16°C for 2 min. Go to Step 2 for 35×, 50°C for 5 min, and 80°C for 20 min. The reaction (5 µL out of 10 µL) was chemically transformed into NEB stable *E. coli*, and then plated on appropriate antibiotic plates.

### Modified plasmids

The following plasmids were created using the isothermal assembly method: BE3_ΔUGI, APOBEC, UDG, Vsr, DNMT, polβsubunit, UvrD, UNG1, UNG2, and MUG. Except for BE3_ΔUGI, which removed UGI from the BE3 plasmid, the other plasmids supplement a DNA repair gene to the existing BE3 plasmid (for details on strains and plasmids, see Table S1). The DNA repair genes were amplified from *Streptomyces* Strain B using primers specific to each target gene (for a list of oligos used in this work, see Table S2). Isothermal assembly was performed as follows: PCR-amplified DNA fragments were combined at a 1:3 backbone-to-insert molar ratio, with a total DNA volume of 5 µL. Next, 5 µL of NEBuilder HiFi DNA Assembly Master Mix was added to bring the final reaction volume to 10 µL. The assembly reaction was incubated at 50°C for 60 min. Following assembly, 5 µL of the reaction mixture was used to transform chemically competent NEB Stable *E. coli* cells. We provide the amino acid sequence of genes tested in Table S3.

### Conjugation in *Streptomyces*

Sequence-verified plasmids were electroporated into the methylation-deficient ET12567/pUZ8002 *Escherichia coli* strain using BioRad Gene Pulser Xcell Electroporation System (BioRad electrocuvette, 1 mm gap; 1.8 kV). Cells were recovered for 1 h in a shaking incubator at 37°C. After this initial recovery, the cells were transferred into a culture tube containing 1 mL of LB supplemented with 2.0 µL apramycin (50 mg/mL), chloramphenicol (25 mg/mL), and kanamycin (50 mg/mL) for a total of 2 mL of culture. The subsequent 2 mL of liquid culture was then grown overnight in a shaking incubator at 37°C. The next day, 1 mL from the overnight culture was added to fresh 4 mL of LB supplemented with antibiotics and 20 mM CaCl_2_ and allowed to grow for 2.5 h in the 37°C shaker. Subsequently, the new culture was pelleted at 3,000 × *g* for 15 min in the Sorvall ST 40R centrifuge. The spent media were carefully decanted into waste, and then the cells were resuspended with TSB media and transferred into a clean, autoclaved 1.5 mL Eppendorf tube. The cells were washed with 1 mL TSB media (30 g/L tryptic soy broth, 100 g/L sucrose, 4 g/L glycine) three times using the Eppendorf centrifuge 5424 for pelleting and a 1 mL pipette for resuspension. The TSB medium washed ET12567/pUZ8002 *E. coli* was then mixed with 25 µL cotton-filtered, concentrated, and germinated *Streptomyces* spores. For *Streptomyces* spore preparation, spores were washed twice with 1.0 mL TSB, centrifuging at 3,000 × *g* for 3 min. Washed *Streptomyces* spores were resuspended in 25 µL TSB × number of conjugation, and then germinated at 42°C for 20 min in a heat block. The mixture was then used to create 5.0 µL *Streptomyces* and *Escherichia coli* biomass spots on solid IWL4 plates supplemented with 60 mM CaCl_2_ (20). The spots on the plate were dried in a laminar flow hood for 30 min; then, the plates were incubated overnight at 30°C. On the next day, the spots were transferred to ISP4 plates supplemented with appropriate antibiotics. Exconjugants were visible on plates between 3 and 6 days.

### Sanger sequencing sample preparation

For colony screen PCR, exconjugants were picked with a pipette tip, and then transferred to 10 µL water (molecular grade) in a PCR tube. The mixture was incubated at 95°C for 10 min, and then spun down for 1 min. Subsequently, the mixture was used in a PCR reaction as a DNA template 1 µL. The PCR reaction was run on one agarose gel electrophoresis, and then the target DNA band was excised, followed by gel purification with the Zymoclean Gel DNA Recovery Kit (catalog no. ZD4008). DNA was eluted from the purification column in 10 µL of Invitrogen UltraPure distilled water. DNA concentration was determined using a NanoDrop spectrophotometer. For Sanger sequencing, 11.2 µL of DNA (3 ng/µL) was combined with 0.8 µL of sequencing primer (25 µM) and submitted to ACGT DNA Sequencing Services. Screening primers were designed to produce PCR amplicons of approximately 700–1,600 bp in length. When necessary, a sequencing primer was designed to bind approximately 200–250 bp upstream of the target region. When necessary, *Streptomyces* genomic DNA was obtained using the Qiagen DNeasy Ultraclean Microbial Kit (catalog no. 12224-50) *Streptomyces* cells grown on a plate or from a 4-day liquid culture.

### Editing verification and sample analysis

Sanger sequencing trace sample files (.ab1) were uploaded to the EditR web tool (21), and the corresponding sgRNA was provided. EditR deconvoluted the fluorescence area of all four bases within the target region to output numerical percent values. The percentage values representing the editing data were manually transferred to Microsoft Excel. Further analysis was performed with a custom Python script. Sanger sequencing generates a non-trivial amount of background noise (base-calls for the non-cognate base at each position) that varies from sample to sample. When viewing the Sanger sequencing results for the entire 20-base spacer sequence, the difference between an edited base and the background is obvious. However, for clear and concise illustration of the results for many sgRNAs in a single figure, we do not show the entire 20-base region. Instead, we plot the base calls for all cytidines within the editing window, and we provide a measure of the background Sanger sequencing noise as the global average variation (GAV). We first generate the arithmetic mean of the summed non-cognate bases called at each position of the 20-base sgRNA spacer sequence not shown in the plot. We then average these means for all sgRNAs compared in a plot. For example, the GAV of 9.51 in Fig. 1d denotes that across all experiments represented in this plot, the background noise of base-calls that did not match the cognate base at each position was 9.51%.

## RESULTS

### Distinct outcomes from cytosine base editors in different *Streptomyces* strains

Initially, we aimed to utilize the published *Streptomyces* codon-optimized CBE (15) to manipulate natural product biosynthesis in two *Streptomyces* strains isolated from disease-suppressive soil. Based on multi-locus sequence alignment, one strain (hereafter designated Strain A) was highly similar to *Streptomyces durhamensis*, and the second (hereafter Strain B) was highly similar to *Streptomyces sporoaveus*. Sequence data for these type strains were retrieved from PubMLST. We designed sgRNAs to generate nonsense mutations in eight genes embedded in natural product biosynthetic gene clusters in Strain A and five in Strain B, with some genes targeted by multiple guides. While Strain A yielded the expected C:G to T:A base edits (Fig. 1d and e and Fig. S1), we surprisingly observed high-frequency C:G to G:C transversion edits in Strain B. This pattern held true across several, but not all, target sites (Fig. 1f and g and Fig. S2). Cumulatively, C:G to G:C mutations comprised 44 of all editing outcomes in Strain B compared to only six in Strain A. Earlier reports of base editing in *Streptomyces* spp. (15, 18) mirror the observations in Strain A, suggesting that Strain B is an outlier. Due to the surprising nature of these results, we decided to investigate the driver of C:G to G:C base editing in Strain B.

### Divergent editing outcomes at identical target sequences

**Fig 2:**
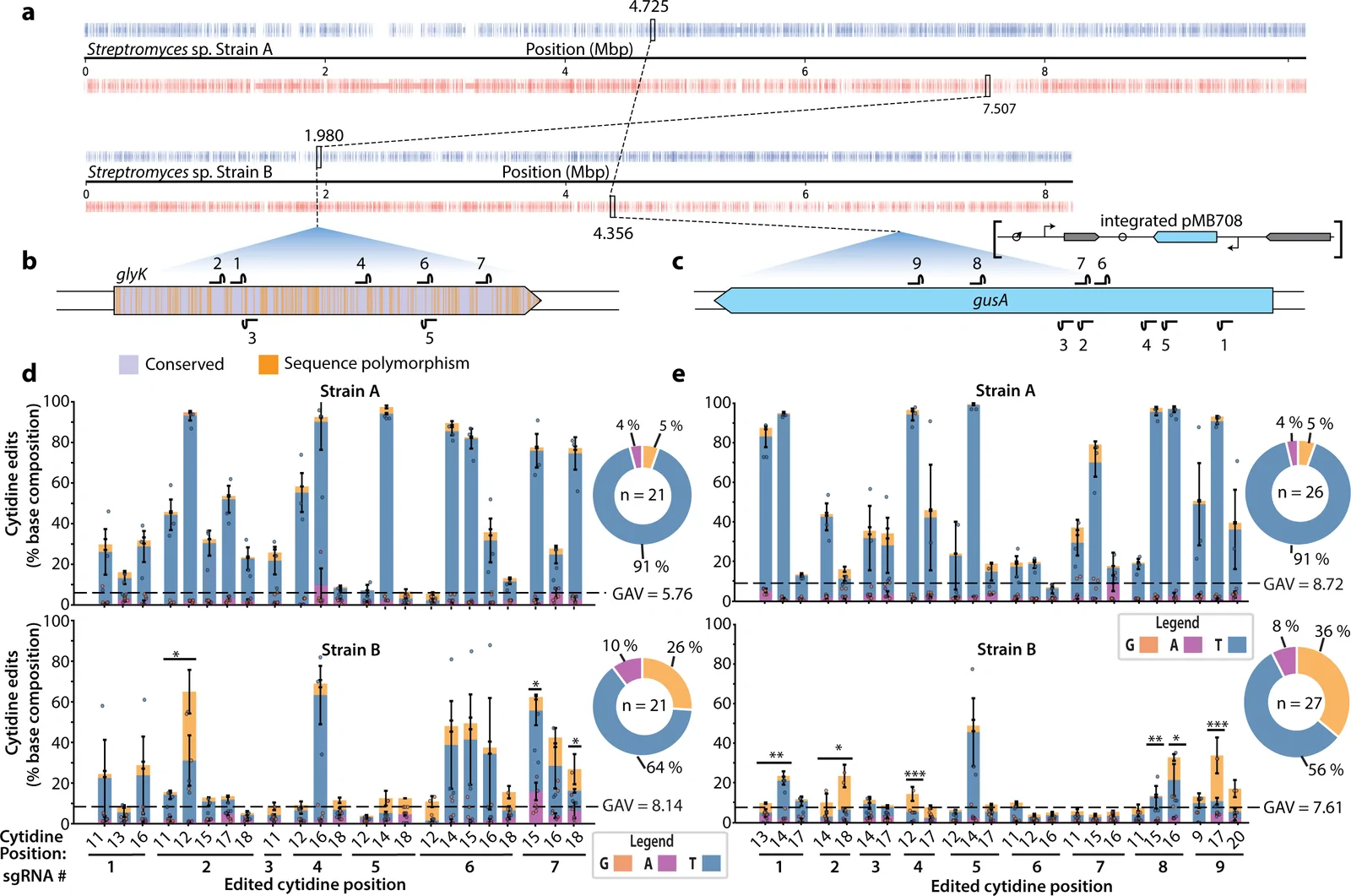
Different cytidine editing outcomes in strains A and B with identical sgRNAs. (**a**) Gene organization on the chromosomes of strains A and B showing CDSs in the forward (blue, top) and reverse (red, bottom) orientations, with boxes highlighting the relative location of the endogenous *glyK* gene (**b**) and the introduced pMB708 sequence (**c**). (**b**) Conserved DNA sequence that could be targeted with sgRNAs is highlighted for *glyK*. (**b and c**) Relative positions of sgRNAs, with numbering corresponding to the bar graphs below, are shown. (**d**) Cytidine editing outcomes across seven sgRNAs targeting *glyK* in strains A (top) and B (bottom). Pie charts at the right of each bar graph show cumulative editing outcomes for all positions that were edited at levels above the global average variance (GAV). (**e**) Similar to (**d**), but for sgRNAs targeting the integrated plasmid pMB708 in each strain. Error bars represent the standard error of the mean (SEM) from three biological replicates, and statistical significance is from the Student *t*-test *P < 0.05, **P < 0.01, and ***P < 0.001.

The absence of consistent C:G to G:C DNA base transversion at each locus in Strain B indicated target-site dependence (Fig. 1f). This could result from a competition of distinct DNA repair mechanisms whose efficiency differs by locus. Therefore, we wanted to rule out the effect of target-site sequence on editing outcome. We pursued two approaches: (i) finding genes with targetable regions of 100% sequence identity between the two strains; and (ii) introducing an identical transgene into each strain that could be targeted for base editing. For the former approach, we designed guides to edit seven identical targets in the gene *glyK*, encoding glycerol uptake kinase. For the latter approach, we introduced the reporter gene *gusA* encoding β-glucuronidase via (ΦC31-mediated DNA integration at the canonical attB site within the pirA gene in each genome and confirmed integration by PCR and sequencing (Fig. S3). Chromosomal location and regions of 100% sequence identity are shown in Fig. 2a and b.

We again observed differential base editing in the two strains (Fig. 2d and e and Fig. S4 and S5), albeit to a lesser degree. For target sequences within the endogenous *glyK* gene, Strain B gave lower editing frequency overall, but, among the bases that were edited, it gave a higher rate of C:G to G:C (26%) than did Strain A (5%). Strain A once again gave robust C:G to T:A editing (91% of edits) compared to 64% in Strain B. Additionally, two sgRNAs (3 and 5) failed to produce editing in Strain B, while Strain A had moderate editing at these exact sites (Fig. 2d). The results observed when targeting the heterologous *gusA* gene in the two strains mirrored those of *glyK*. There was both an overall decrease in editing efficiency in Strain B and a higher proportion of C:G to G:C transversion mutations at the sites that were edited (Fig. 2e) and (Fig. S6 and S7). Taken together, these results indicated that local genetic context was not solely responsible for the increased transverse base editing observed in Strain B versus Strain A. We hypothesized that the distinct editing outcomes were due to system-wide differences, for example, in DNA repair proteins.

### Delayed onset of BE activity in Strain B

**Fig 3:**
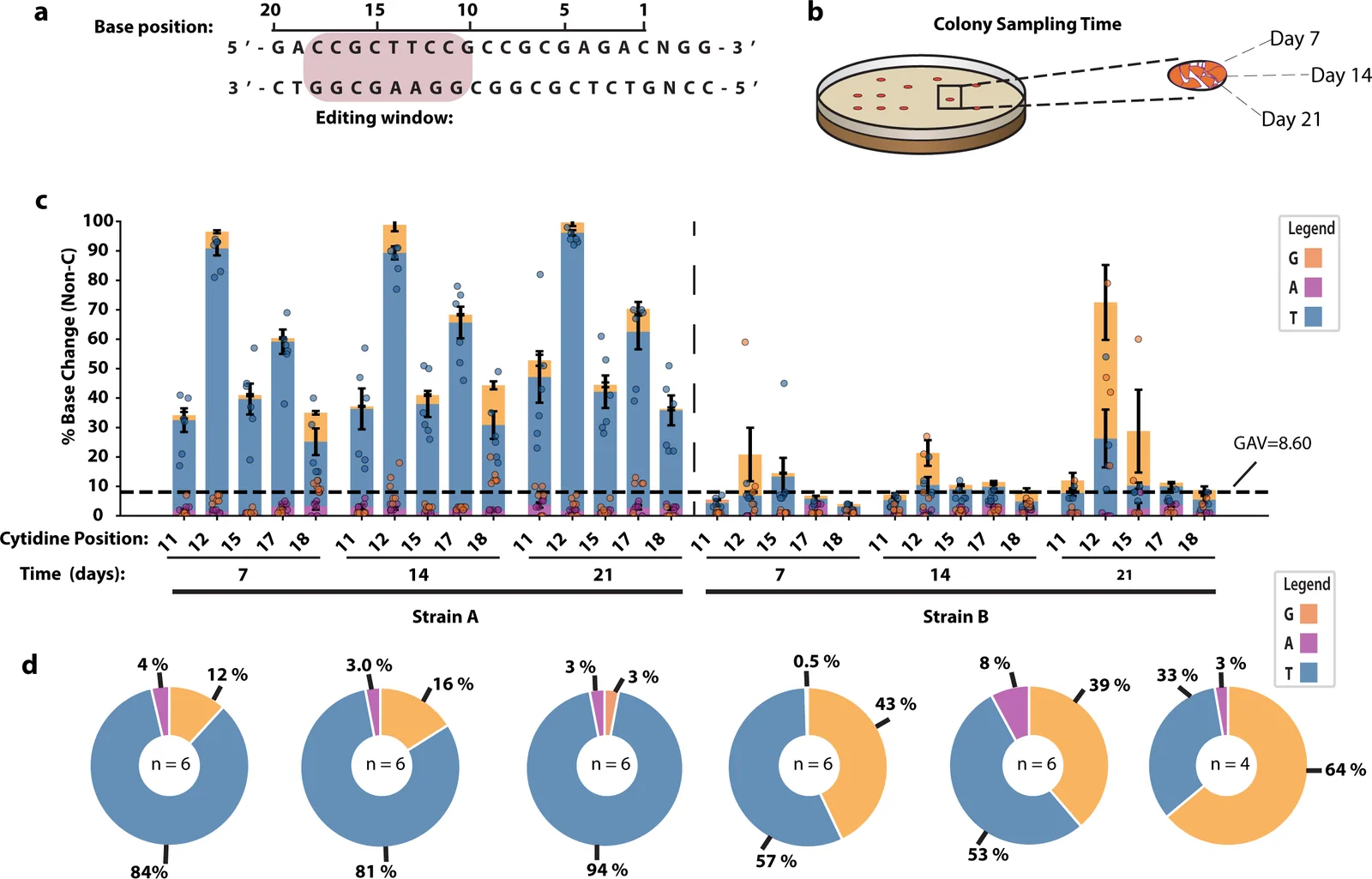
Delayed onset of the base editing activity in Strain B. (**a**) Shared sgRNA used in this experiment with the editing window shaded. (**b**) Schema of the repeated colony sampling strategy. Three colonies per plate sampled at three time intervals across two independent biological replicates. (**c**) Base editing results at days 7, 14, and 21 for strains A and B. (**d**) Overall base editing outcomes at each time interval. Plates were kept at 30°C for the duration of the experiment. The error bar is the SEM, and GAV is calculated as previously described.

We noticed that Strain B generally took longer to show BE activity compared to Strain A. We tested this systematically using *glyK* sgRNA #2. Specifically, we targeted strains A and B with identical base editing reagents and sampled cells from single colonies after 7, 14, and 21 days (Fig. 3). We observed rapid C:G to T:A transition editing in Strain A at the earliest timepoint (day 7) (Fig. 3c and d). There was no statistical difference in editing outcomes when cells from the colony were resampled at days 14 and 21. In contrast, we observed very low editing in Strain B until the 21-day timepoint. At day 21, editing at base 12 in the target sequence jumped from 20 to 70%, and C:G to G:C transversion mutations were the most frequent edit observed (Fig. 3c and d). When streaked for single colonies on the ISP4 solid medium, Strain B appears to have produced aerial hyphae after 2 days, whereas Strain A has not (Fig. S8).

### Identifying genetic differences between strains A and B

**Fig 4:**
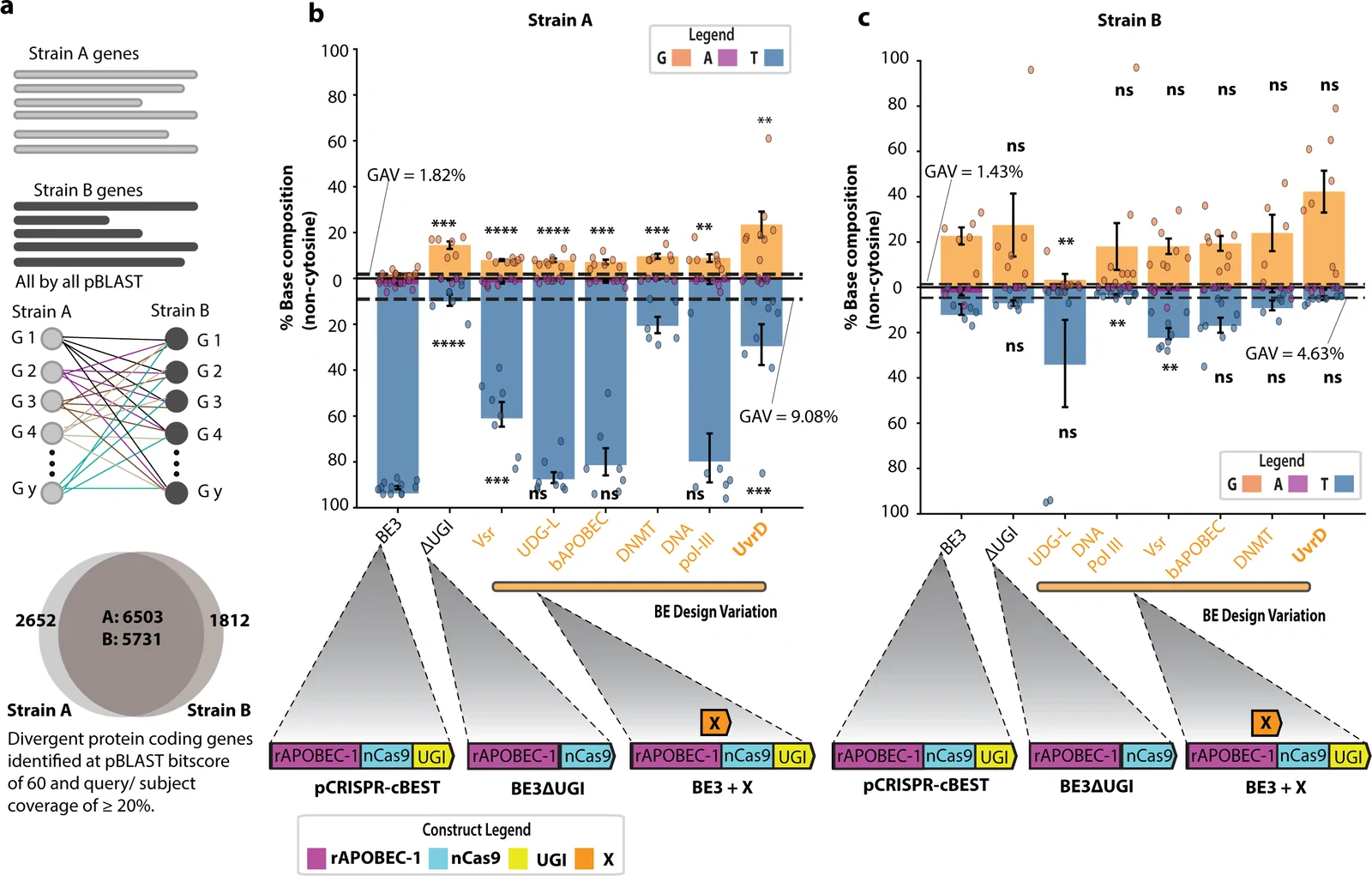
DNA repair genes from *Streptomyces* Strain B affect base editing outcome in *Streptomyces* Strain A. (**a**) All-by-all NCBI protein BLAST search comparing the two whole genomes at a 60-amino acid cutoff. A one-way, best-hit divergence analysis was utilized to generate the diagram. The total number of genes in each organism is shown. (**b and c**) Putative DNA repair genes identified from Strain B constitutively expressed in strains A and B, respectively. Multiple independent plasmid constructs were assembled. Coding DNA sequence X represents an auxiliary protein added in each construct, and these are labeled on the *x*-axis. Base editing results in strains A and B with focal position 12 PAM-distal. The error bar is the standard error of the mean across three independent biological replicates and three colonies per biological replicate. Welch’s *t*-test was used for significance *P < 0.05, **P < 0.01, and ***P < 0.001.

We hypothesized that genetic differences between the two strains, particularly in genes putatively involved in DNA repair pathways, influenced their unique editing outcomes. The BE3 multidomain complex induces two primary types of DNA damage: cytidine deamination resulting in a U:G mismatch, and single-strand breaks on the sgRNA complementary DNA strand. These DNA lesions are predominantly repaired through base excision repair and DNA replication processes, which are mediated in part by mismatch repair enzymes, base excision repair enzymes, and DNA polymerases (22). Consequently, we focused on differences in encoded enzymes capable of generating abasic sites, such as uracil DNA glycosylases, as well as translesion DNA polymerases. We performed an all-by-all protein BLAST comparison between the two genomes (Fig. 4a). The amino acid identities between each gene from Strain B compared to their closest homolog in Strain A showed a bimodal distribution. There is one peak at approximately 80% identity and a second peak at approximately 35% identity (Fig. S9a and b). We interpret this as indicating that the genes shared in the last common ancestor of strains A and B that have diverged by orthology are on average 80% identical, and the second group representing genes that have been added via horizontal gene transfer since the divergence of strains A and B. Sequence homology and BLAST bitscore are correlated (Fig. S9c through f), and the latter was used to identify genes that were unique to each strain (Fig. 4a). We used a protein BLAST bitscore cutoff of 60 with ≥20% query and subject coverage to identify genes lacking high-confidence homologs between the two strains. By this criterion, approximately 1,800 proteins in Strain B did not have a highly similar counterpart in Strain A.

Among the encoded proteins unique to Strain B is a very-short-patch repair (vsr) protein (23–25), (putatively involved in T:G mismatch repair), an ATP-binding protein annotated as a DNA polymerase III delta prime subunit, and DNA (cytosine-5)-methyltransferase 1 (DNMT). Additionally, the superfamily I DNA-RNA helicase UvrD gene was of particular interest, as UvrD is known to participate in nucleotide excision repair and mismatch repair through interactions with UvrC and MutH, respectively (26, 27). In addition to the divergent UvrD, Strain B also possessed two more UvrD ATP-dependent DNA helicase UvrD/PcrA-related enzymes that shared 25 and 26% amino acid identity to the divergent homolog in Strain B. Importantly, Strain A also had these two enzymes at 27 and 30% amino acid identity but lacked the divergent UvrD enzyme.

We also identified a gene computationally annotated as a uracil DNA glycosylase/O-acetyl-ADP-ribose deacetylase, hereafter referred to as the UDG-like or UDG-L gene. The encoded protein has an N-terminal leucine aminopeptidase domain, a central UDG domain, and a C-terminal NAD:Arginine ADP-ribosyltransferase domain according to automated protein domain annotation by InterProScan. While this UDG-L gene is specific to Strain B, both strains A and B contain a set of uracil glycosylase genes canonically found in bacterial genomes: UNG1, UNG2, and the mismatch-repair specific MUG orthologs. UDG-L is present in the same genomic region as the aforementioned UvrD. We included this UDG-L gene in our analysis because of the important role of base-excision repair in the outcome of base editing.

Motivated by these findings, we engineered constructs expressing the putative DNA repair proteins unique to *Streptomyces* Strain B in the editing plasmid (Fig. 4b) to test whether introducing these proteins to *Streptomyces* Strain A would recapitulate the base editing outcomes observed in Strain B.

### DNA repair proteins from *Streptomyces* Strain B affect outcomes of base editing in *Streptomyces* Strain A

Previous reports of engineered transversion BEs have heavily relied on CBEs lacking UGI. We compared the editing outcomes of BE3 from pCRISPR-cBEST with a ΔUGI version and several editing constructs that contained a second expression cassette encoding a Strain B-specific putative DNA repair enzyme. These were tested in strains A and B. To more clearly compare both the losses of C:G to T:A editing and C:G to G:C editing across these constructs, we plotted data with the transition edits plotted below the *y* = 0 axis and transversion edits above (Fig. 4b and c).

The importance of uracil DNA glycosylase activity is readily apparent. Deleting the UGI domain from BE3 abrogated most of the C:G to T:A activity in Strain A while increasing C:G to G:C transversion mutations to levels similar to those seen in Strain B. Leaving UGI intact but adding a putative repair enzyme from Strain B led to partial or full retention of C:G to T:A transition editing events and a moderate but statistically significant increase in C:G to G:C transversions. For Vsr, UDG-L, bAPOBEC, DNMT, and Polβ subunit, the increase in transversion edits was less than that seen in the ΔUGI construct. Adding DNA:RNA Helicase UvrD resulted in the greatest increase in transversion mutations.

In Strain B, deleting UGI had no impact on the level of transversion edits. The UGI-competent BE3 editing outcome closely resembles the behavior of the ΔUGI construct in Strain A, perhaps suggesting that UGI is unable to functionally inhibit the enzyme involved in uracil excision in Strain B. Interestingly, overexpression of UDG-L substantially reduced transversion mutations and increased transition mutations, akin to the expected behavior of CBEs in other organisms. Overexpression of UvrD yielded the greatest level of C:G to G:C transversion mutations, although large variance in the measurement prevents us from rejecting the null statistical hypothesis that this change is due to random chance.

While Fig. 4 only reports editing efficiency on the most commonly edited position within the editing window, stark differences exist between strains A and B at other cytidines in this window (Fig. S10, Fig. S11, and Fig. S12) for editing window and Sanger base call data, respectively. Strain A yielded frequent edits at positions 11, 12, 15, 17, and 18 (PAM-distal) for BE3 and most of the alternative constructs tested in Fig. 4. The two constructs that gave the highest frequency of C:G to G:C transversion mutations at position 12 (BE3-ΔUGI and UvrD), each had low-frequency mutations at other positions. In contrast, Strain B showed selective editing at position 12, except for a single outlier with high editing at position 11 in the UDG construct (Fig. S10b). Additionally, we preliminarily evaluated Ung1, Ung2, and MUG from Strain B in Strain A but did not observe an increase in C:G-to-G:C editing frequencies (Fig. S13). Notably, Ung1 reduced C:G to T:A editing, whereas Ung2 increased C:G to T:A editing at positions 12 and 15. In contrast, MUG produced moderate levels of C:G to T:A editing only at position 15.

**Fig 5:**
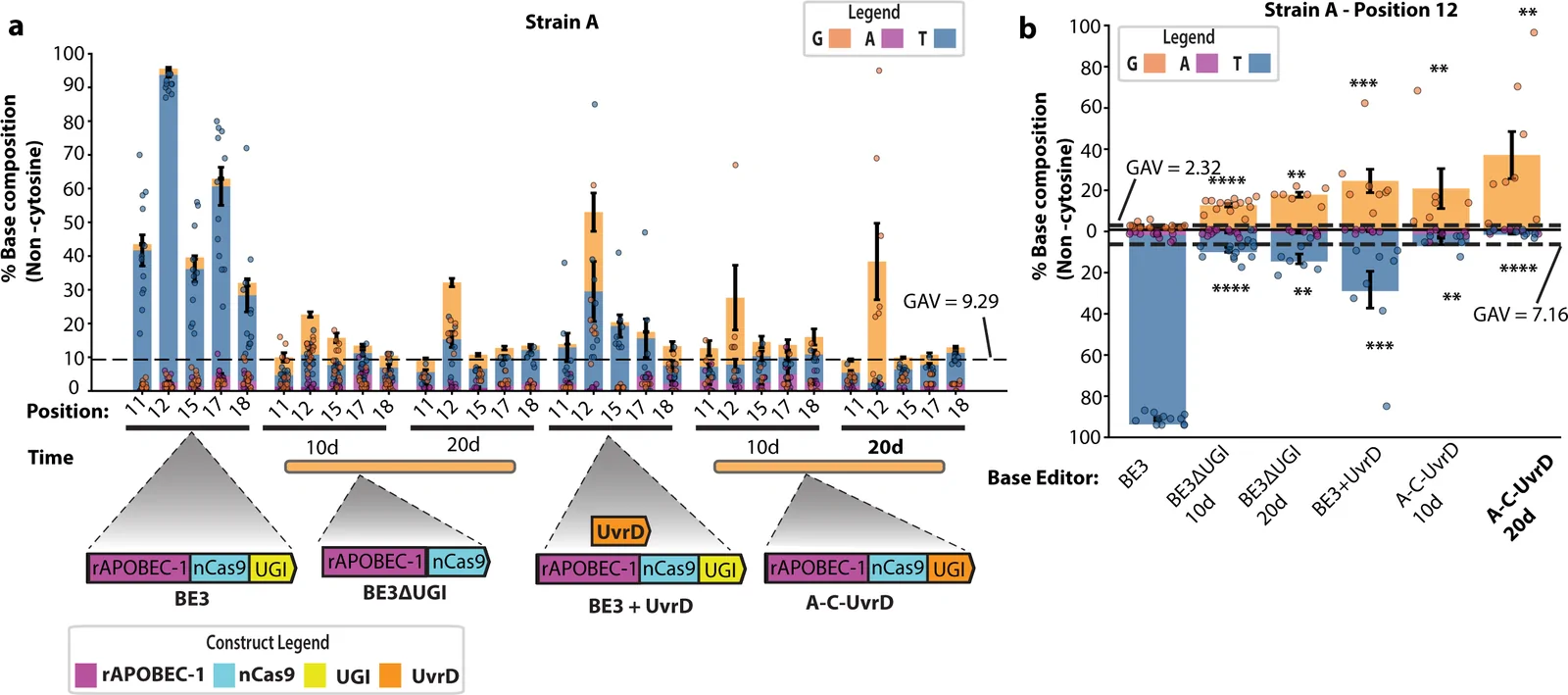
*DNA helicase UvrD* promotes reduced bystander editing in *Streptomyces* Strain A. Panels **a** and **b** show the constructs BE3 WT, A-C-ΔUGI, BE3 + UvrD (UvrD under a constitutive promoter and expressed somewhere else on the base editing plasmid), and A-C-UvrD (rAPOBEC-nCas9 fused to UvrD). The time when samples were analyzed is shown in days. The same sgRNA was used in all the constructs. (**a**) Base editing results are shown across the full editing window. (**b**) Base editing results at position 12 of the editing window. C to G is shown at the top and C to A or T at the bottom. Error bars represent the SEM, and the statistical significance is from the Mann-Whitney test. *P < 0.05, **P < 0.01, and ***P < 0.001.

Because high-efficiency transversion base editors could be useful genetic tools, we attempted to combine elements that favored C:G to G:C edits in a single fusion protein. We fused UvrD to a ΔUGI BE3, giving a final domain composition of rAPOBEC-1::nCas9::UvrD. This was introduced to Strain A with the same sgRNA used to compare divergent DNA repair enzymes in Fig. 4. The resulting construct yielded the greatest combination of selectivity for position 12 (Fig. 5a) and C:G to G:C transversion edits (Fig. 5b) compared to BE3, BE3-ΔUGI, or BE3 + UvrD (as an independently expressed protein). It is also noteworthy that the transition editing (C:G to T:A) seen for this construct is sufficiently low that the baseline of sequencing noise can account for all transition mutations observed.

### Dinucleotide effect on editing frequency

We investigated whether dinucleotide sequence context influenced editing outcome in strains A and B using the Kruskal-Wallis test (Fig. S14). No significant association was detected between the upstream dinucleotide (NC) context and the editing frequencies of C to G, C to T, or C to A (all *P* > 0.05) (Fig. S14a). In contrast, a significant association was observed between downstream dinucleotide (CN) context and C to T editing frequencies (Kruskal-Wallis, H = 18.51, *P* = 0.000345). Dunn’s post-hoc test with Holm correction showed that targets with a downstream CG context exhibited significantly lower C to T editing frequencies than targets with either CC (adjusted *P* = 0.0105) or CT (adjusted *P* = 0.00065) context, whereas no other pairwise comparisons were significant (Fig. S14b). These findings suggest that, in Strain A, the downstream nucleotide may influence the C to T editing outcome, with greater editing when the target base is followed by a pyrimidine. We next examined the influence of dinucleotide sequence context on the editing outcome in Strain B (Fig. S14c and d). No statistically significant associations were detected between either upstream (NC) or downstream (CN) dinucleotide context and the frequencies of C to G, T, or A editing (Kruskal-Wallis, all *P* > 0.05). These findings suggest that within the statistical power of the present data set, dinucleotide sequence context did not measurably influence editing outcome in Strain B.

## DISCUSSION

This work began with a surprising observation that an identical CBE tool produced strikingly different editing outcomes in two strains within the genus *Streptomyces*. Strain A produced the expected transition (C:G to T:A) mutations in high frequency, and Strain B produced high-frequency transversion (C:G to G:C) edits at some sites (Fig. 1). This difference was reproduced when identical DNA sequences were targeted in the two strains (Fig. 2). Many factors have been reported to impact base editing efficiency, including target DNA topology, the dynamics of nCas9 searching for the PAM, the binding kinetics in the 20-bp RNA complementary sequence, and GC content of different genomes (28). In addition, within the base editing window, the base upstream or downstream of the target base can influence editing efficiency (4).

The unique editing outcomes between strains A and B could be the result of (i) differential biochemical activity of the BE3 editing tools, or (ii) differences in the DNA repair pathways between the two strains that resolve the initial cytidine deamination to produce uracil. The first option is unlikely. rAPOBEC-1 requires zinc ions for catalysis (29), but this is expected to be available in both strains. No other cofactors are required for catalysis. Thus, differences in DNA repair pathways are the most likely cause of the unique editing outcomes between strains A and B.

Data presented in (Fig. 4) suggest a difference in the activity of uracil DNA glycosylase in the two strains. Strain A shows the expected behavior of BE3 and BE3ΔUGI. Deleting the inhibitor abolishes most of the transition mutation efficiency and renders an editor that equally generates C to G and C to T mutations (1, 14). The similar results observed with BE3 and BE3ΔUGI in Strain B suggest that the uracil DNA glycosylase in Strain B is resistant to inhibition by UGI, or that it was not functional in the first place. Interestingly, we saw a high level of transition mutations when an alternative uracil DNA glycosylase-like (UDG-L) enzyme was overexpressed in Strain B, but this result was not robust (it was only seen in two of five replicates). Future follow-up characterization of the UDGs in Strain B would be needed to better understand this result.

Differences in UDG activity do not explain the boost in transversion mutations observed when UvrD was overexpressed either as a separate protein (Fig. 4) or as a C-terminal fusion to the base editor (Fig. 5). UvrD is annotated as a DNA-RNA helicase, which could contribute to a potential mechanism hypothesized below.

The last observation that contributes to our mechanistic hypothesis is the distinct timing of base editing in the two strains (Fig. 3). Transition mutations in Strain A are observed at our earliest timepoint (7 days after plating), which is approximately when the colony size becomes sufficiently large to harvest cells. Transversion mutations (and indeed any mutations) in Strain B are very low frequency until 21 days after plating. *Streptomyces* spp. have a developmental lifecycle that is well characterized (30). Initially, mycelial cells grow by tip extension. Each of these elongated cells contains multiple copies of the genome comprising a long, linear chromosome. Later in development, elongated polyploid aerial hyphae septate (divide) into small cells that further develop into spores. This septation isolates individual chromosomes. The late occurrence of transversion editing in Strain B may be driven by high rates of homology-directed repair (HDR) of lesions in polyploid cells (restoring lesions to the wild-type sequence). Upon septation of hyphae into monoploid cells, HDR is no longer possible, as there is not a second chromosome that can be used as a repair template, and transversion mutations result. Additionally, our measurement relies on Sanger sequencing rather than next-generation sequencing (NGS). Therefore, the reported editing is the total editing that Sanger sequencing detects rather than the individual sequencing reads provided by NGS.

For a parsimonious explanation that includes our UvrD results, we postulate that overexpression of the DNA-RNA helicase, UvrD, accelerates the melting of the R-loop and dissociation of the nCas9 complex at the target site. If the presence of the nCas9 complex at the target site is one of the intracellular signals that triggers HDR in polyploid cells (31), then dissociation of this complex would limit reversion to wildtype and allow more lesions to persist to the point where they can be eventually converted to stable mutations via the activity of a DNA polymerase.

Testing this model will require many additional experiments that are beyond the scope of this paper. The significance of these studies would depend, in part, on whether the high-transversion mutation activity of the BE-UvrD fusion translates to other organisms, such as human cell lines. These studies are underway. At a minimum, the results presented here provide a new tool for catalyzing C:G to G:C transversion mutations in *Streptomyces* spp. If this translates to human cells, this tool would have a broader impact in creating or correcting transversion mutations for biomedical research or therapeutics.

## SUPPLEMENTAL MATERIAL

Supplemental material jb.00241-26-s0001.pdf Figs. S1 to S14; Tables S1 to S3.

ASM does not own the copyrights to Supplemental Material that may be linked to, or accessed through, an article. The authors have granted ASM a non-exclusive, world-wide license to publish the Supplemental Material files. Please contact the corresponding author directly for reuse.
