# Supplementary material for: Strain-specific outcomes of cytosine-base editing in Streptomyces
Source: J Bacteriol. 2026 Sep 18;208(10):e00241-26. doi: 10.1128/jb.00241-26 (PMC13689562; doi:10.1128/jb.00241-26)
Supplement: Supplementary file 1 [file jb.00241-26-s0001.pdf]

Supplementary Material: Strain-specific outcomes of cytosine-base editing in *Streptomyces*

Maxime M. Boneza[1], **Thomas Keller**[2], **Josephine Mostek**[2], **and Michael J. Smanski**[1,2]

[1]Department of Biochemistry, Molecular Biology, and Biophysics
[2]Biotechnology Institute, University of Minnesota, Saint Paul, MN 55108

Correspondence: msmanski@umn.edu

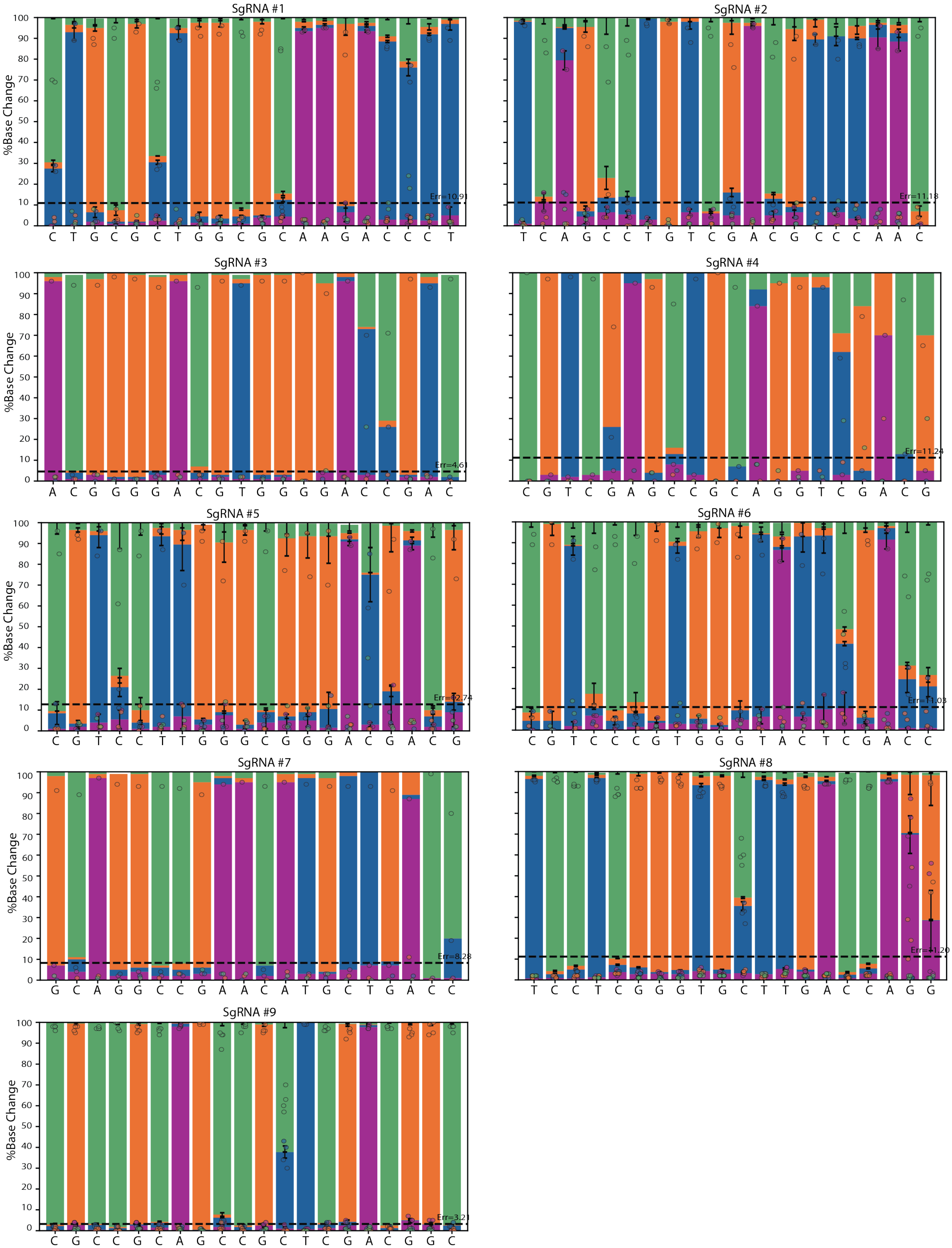

Supplementary Fig. 1. Complete base-editing dataset for *Streptomyces* Strain A. Base-calling frequencies derived from Sanger sequencing chromatograms are shown for each of the nine sgRNA target sites evaluated in *Streptomyces* Strain A. The complete protospacer sequence for each sgRNA is displayed in the 5′–3′ orientation. The dashed line indicates the global average variation (GAV) calculated for each sgRNA target, and error bars represent the standard error of the mean (SEM).

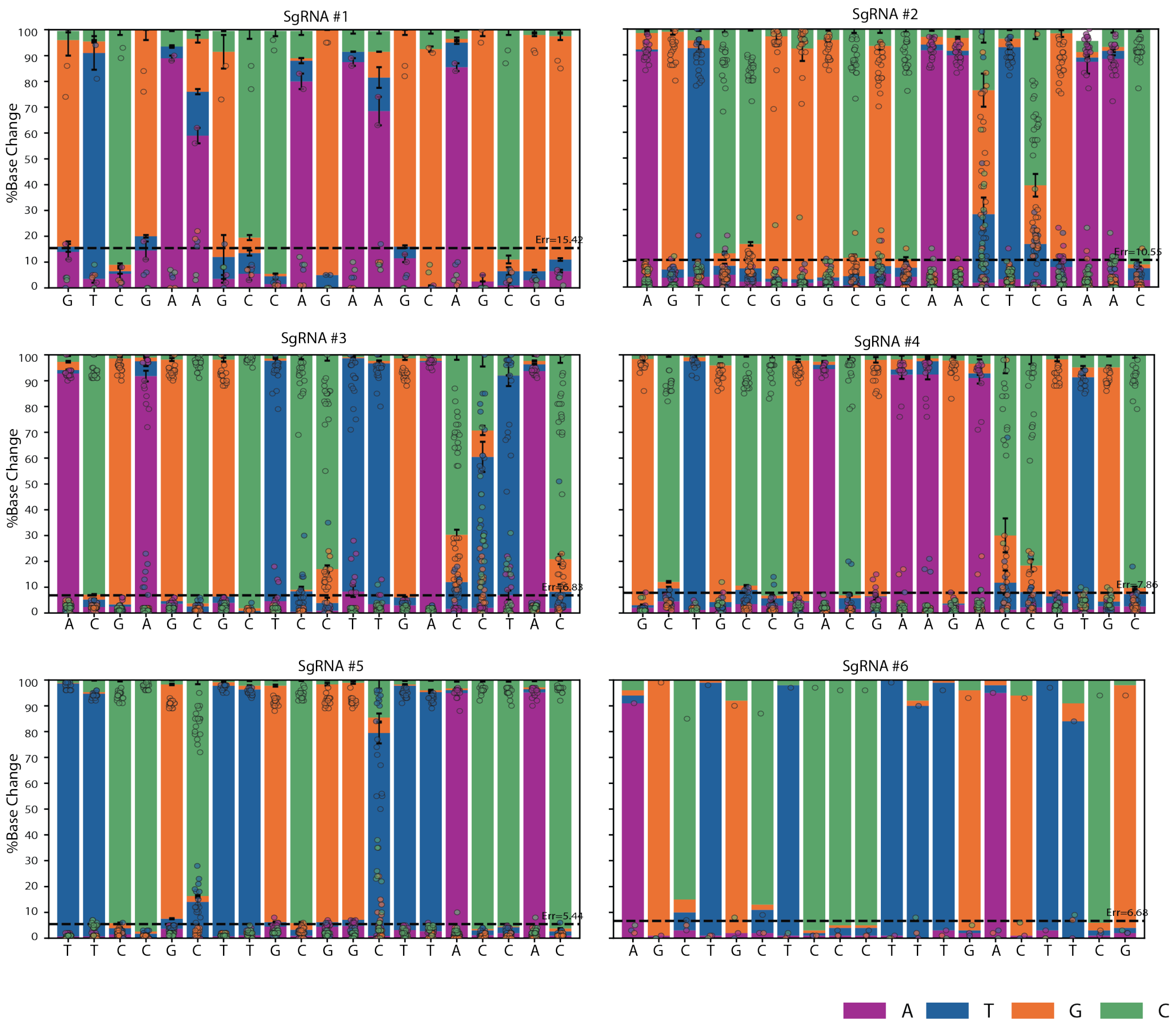

Supplementary Fig. 2. Complete base-editing dataset for *Streptomyces* Strain B. Base-calling frequencies derived from Sanger sequencing chromatograms are shown for each of the seven sgRNA target sites evaluated in *Streptomyces* Strain B. The complete protospacer sequence for each sgRNA is displayed in the 5′–3′ orientation. The dashed line indicates the global average variation (GAV) calculated for each sgRNA target, and error bars represent the standard error of the mean (SEM).

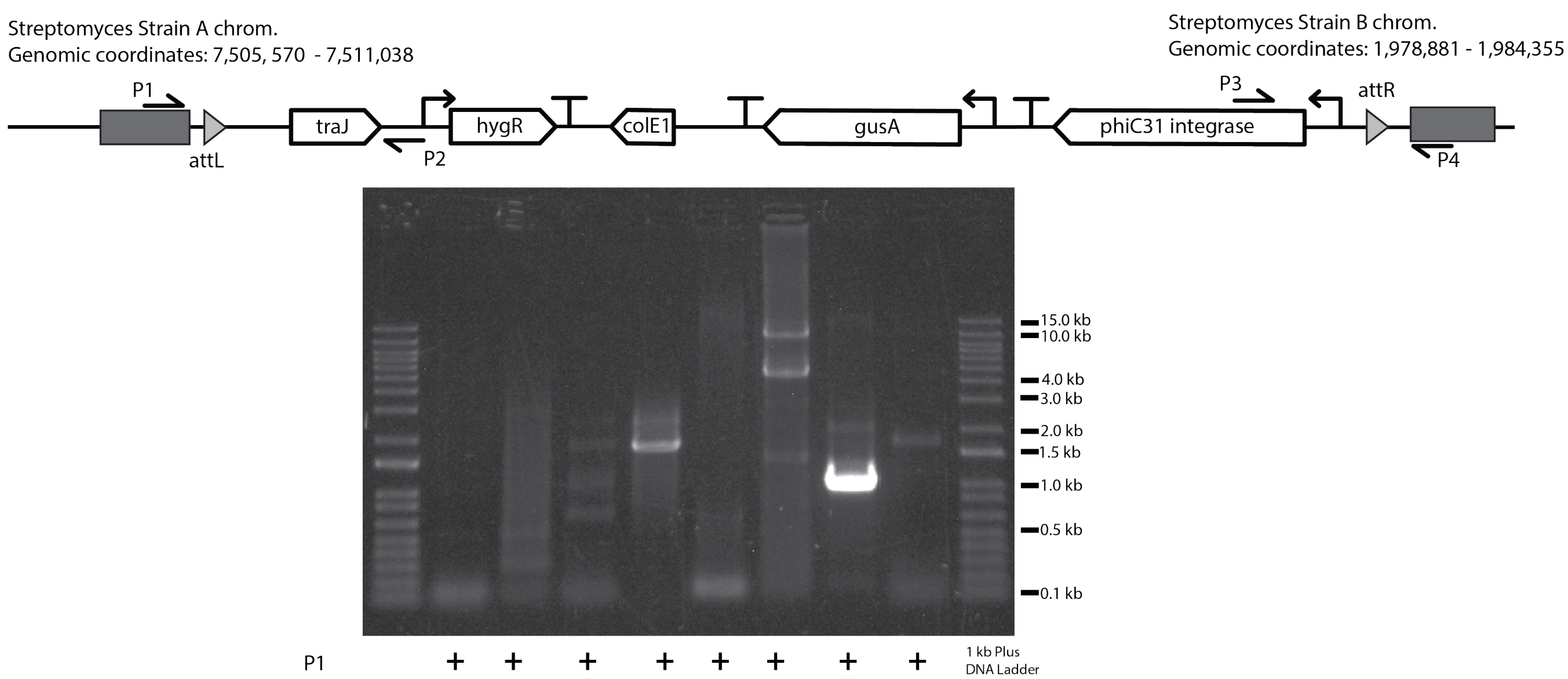

P1	+	+	+	+	+	+	+	+
P2	-	-	+	-	-	-	+	-
P3	-	-	-	+	-	-	-	+
P4	+	+	-	-	+	+	-	-
Strain_A	+	-	-	-	-	-	-	-
Strain_B	-	-	-	-	+	-	-	-
Strain_A_gusA	-	+	+	+	-	-	-	-
Strain_B_gusA	-	-	-	-	-	+	+	+

Supplementary Fig. 3. ΦC31 mediated plasmid integration. a. A map of pMB708 after integration into the attB site. b. Both *Streptomyces* Strain A and B gained resistance to hygromycin. Using primer pairs shown in the table, we observed PCR amplification of expected fragments from each engineered *Streptomyces*. The larger 9kb fragment was sent for sequencing at Plasmidsaurus and showed further confirmed the plasmid integration. Gel run on 1 % agarose gel.

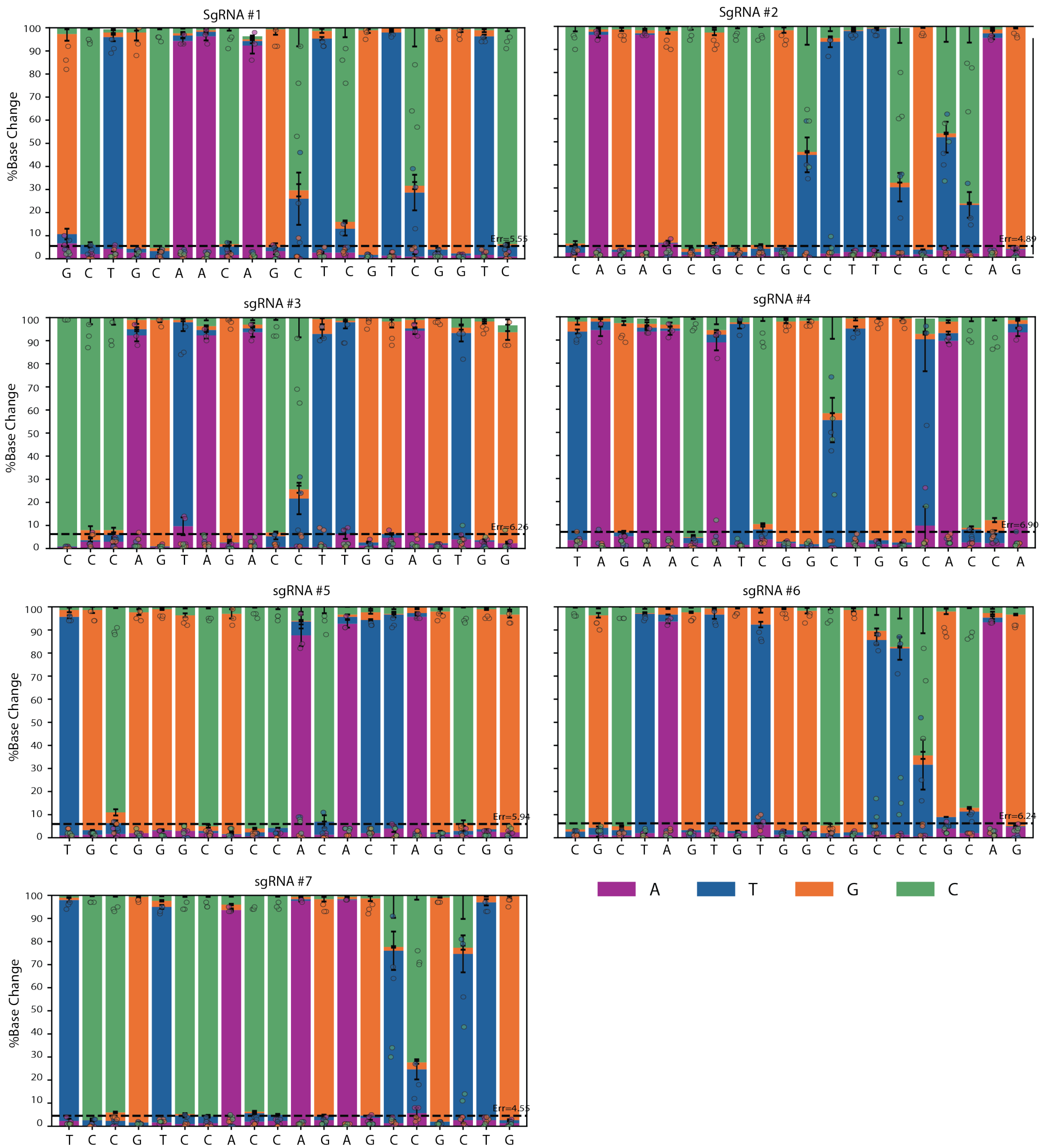

Supplementary Fig. 4. Complete base-editing dataset for *Streptomyces* Strain A glycerol uptake kinase. Base-calling frequencies derived from Sanger sequencing chromatograms are shown for each of the seven sgRNA target sites evaluated in *Streptomyces* Strain A. The complete protospacer sequence for each sgRNA is displayed in the 5′–3′ orientation. The dashed line indicates the global average variation (GAV) calculated for each sgRNA target, and error bars represent the standard error of the mean (SEM).

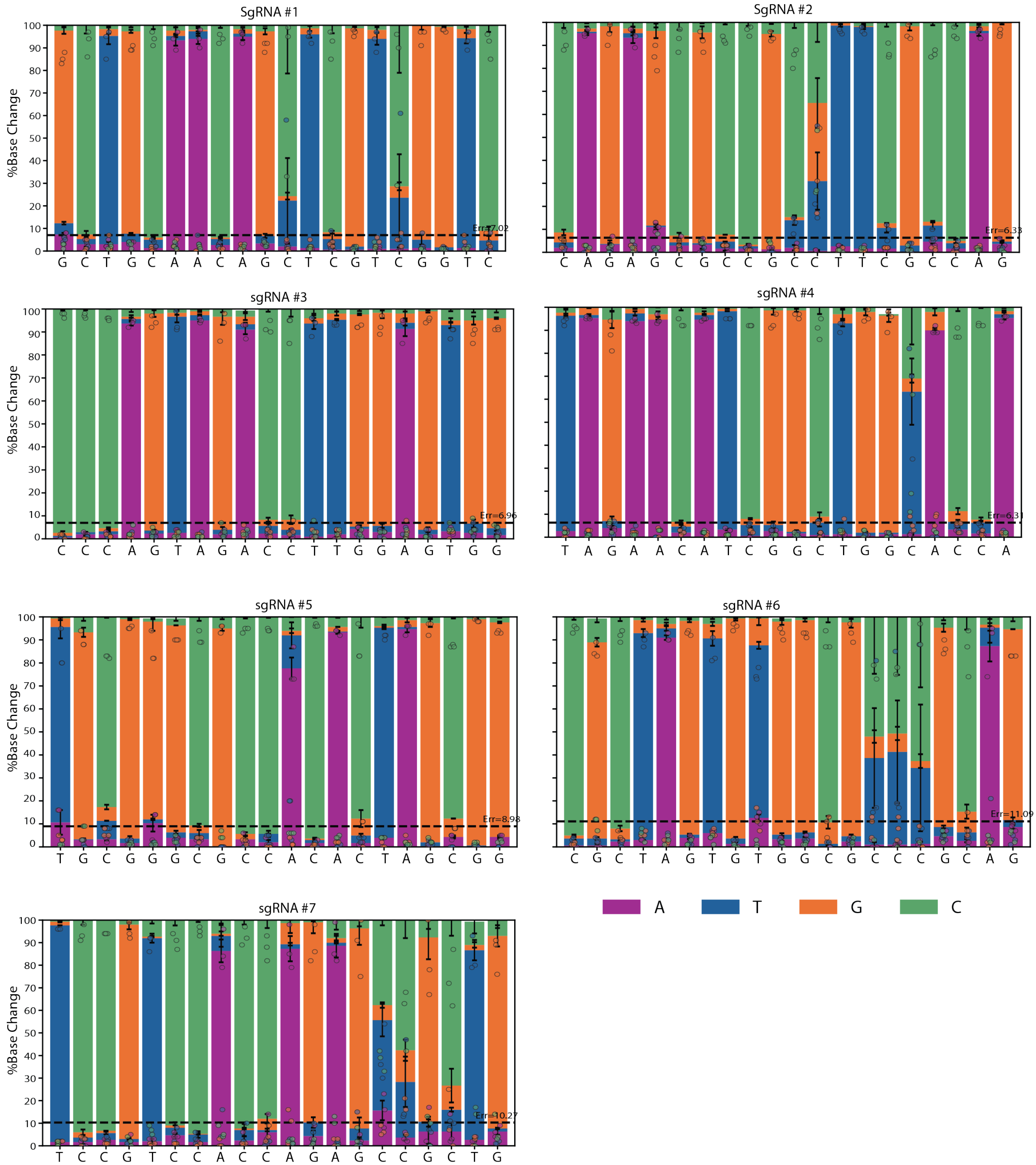

Supplementary Fig. 5. Complete base-editing dataset for *Streptomyces* Strain B glycerol uptake kinase. Base-calling frequencies derived from Sanger sequencing chromatograms are shown for each of the seven sgRNA target sites evaluated in *Streptomyces* Strain B. The complete protospacer sequence for each sgRNA is displayed in the 5′–3′ orientation. The dashed line indicates the global average variation (GAV) calculated for each sgRNA target, and error bars represent the standard error of the mean (SEM).

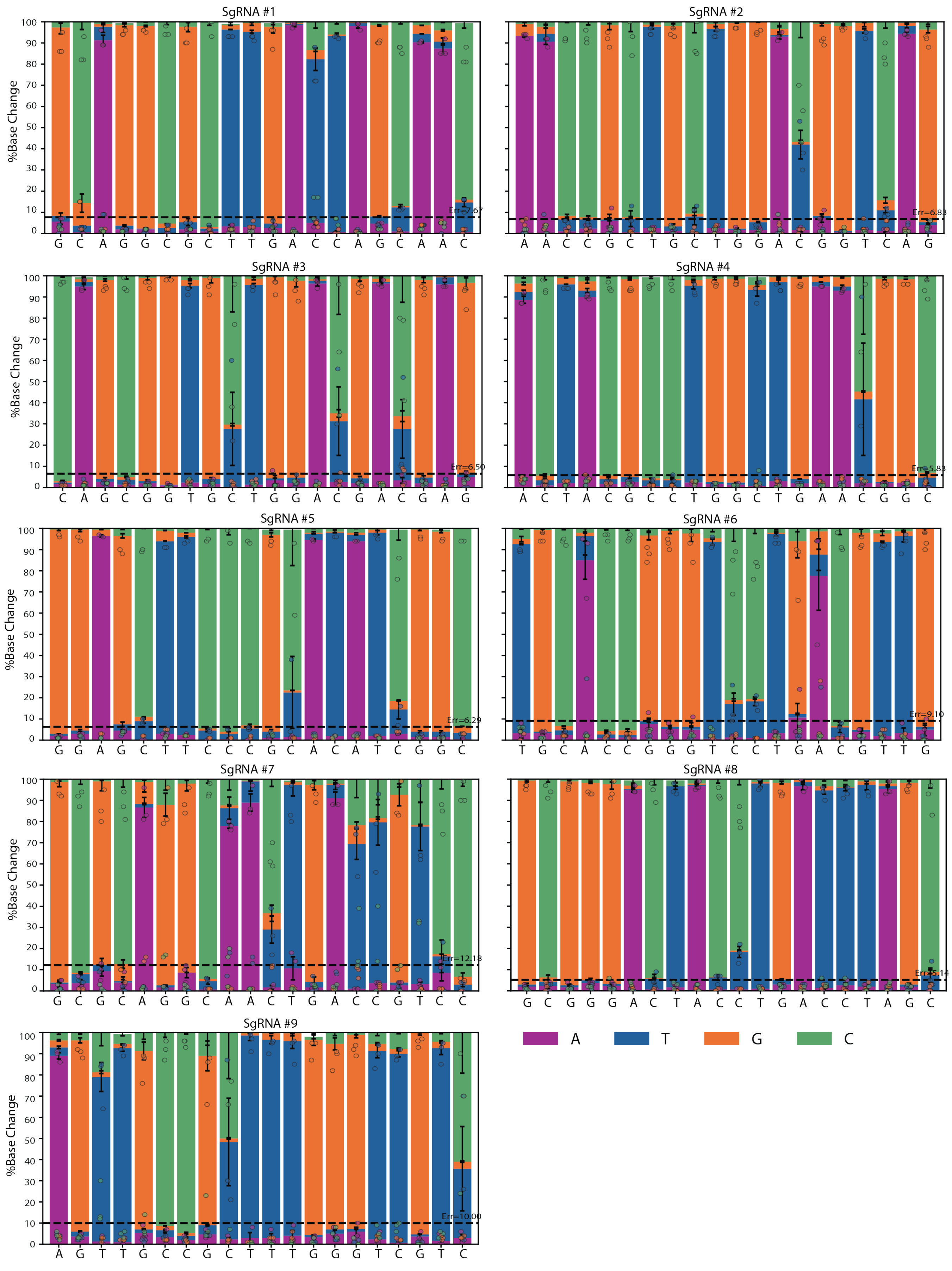

Supplementary Fig. 6. Complete base-editing dataset for Engineered *Streptomyces* Strain A targeting β-glucoronidase. Base-calling frequencies derived from Sanger sequencing chromatograms are shown for each of the nine sgRNA target sites evaluated. The complete protospacer sequence for each sgRNA is displayed in the 5′–3′ orientation. The dashed line indicates the global average variation (GAV) calculated for each sgRNA target, and error bars represent the standard error of the mean (SEM).

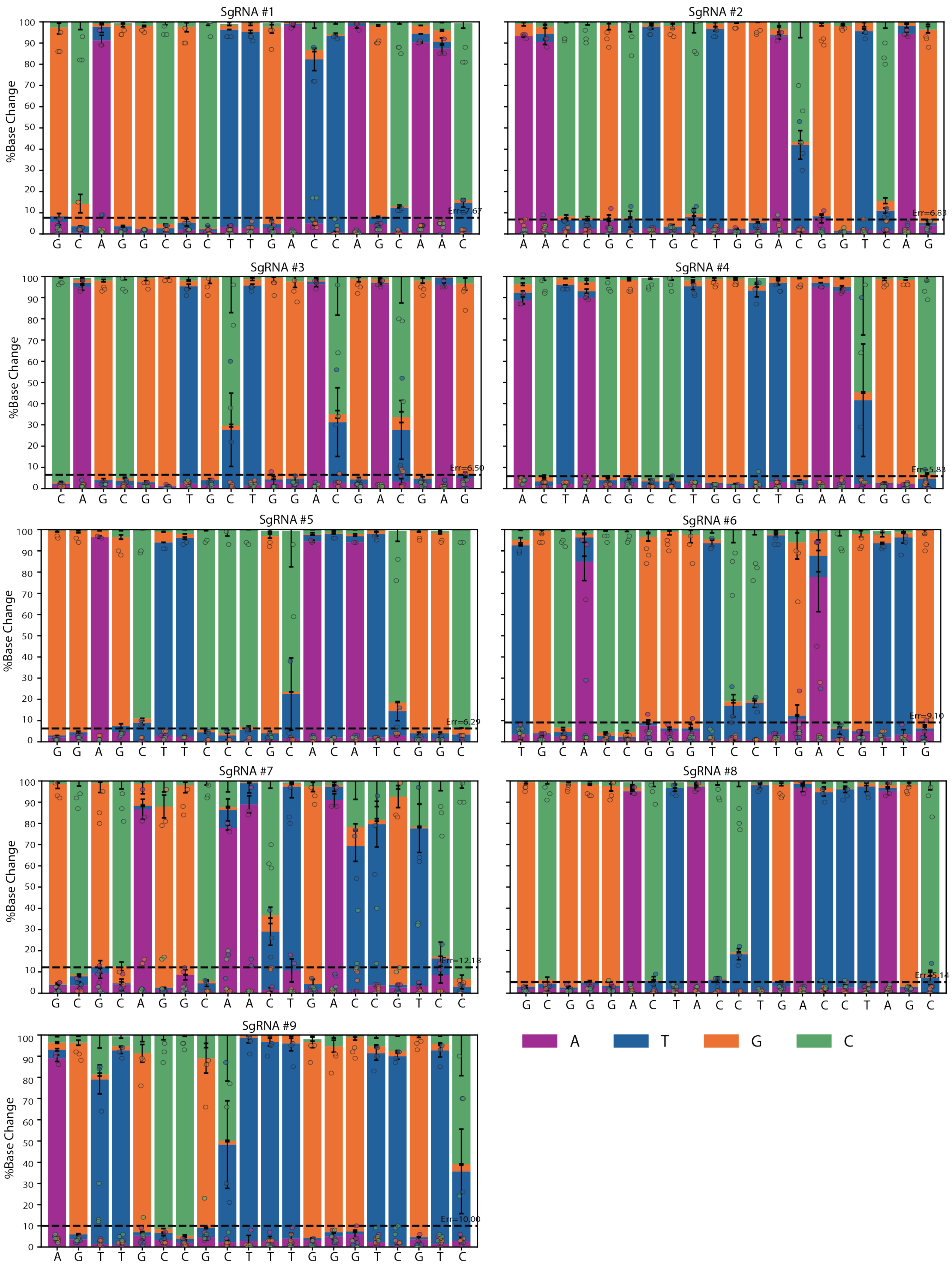

Supplementary Fig. 7. Complete base-editing dataset for Engineered *Streptomyces* Strain B targeting β-glucoronidase. Base-calling frequencies derived from Sanger sequencing chromatograms are shown for each of the nine sgRNA target sites evaluated. The complete protospacer sequence for each sgRNA is displayed in the 5′–3′ orientation. The dashed line indicates the global average variation (GAV) calculated for each sgRNA target, and error bars represent the standard error of the mean (SEM).

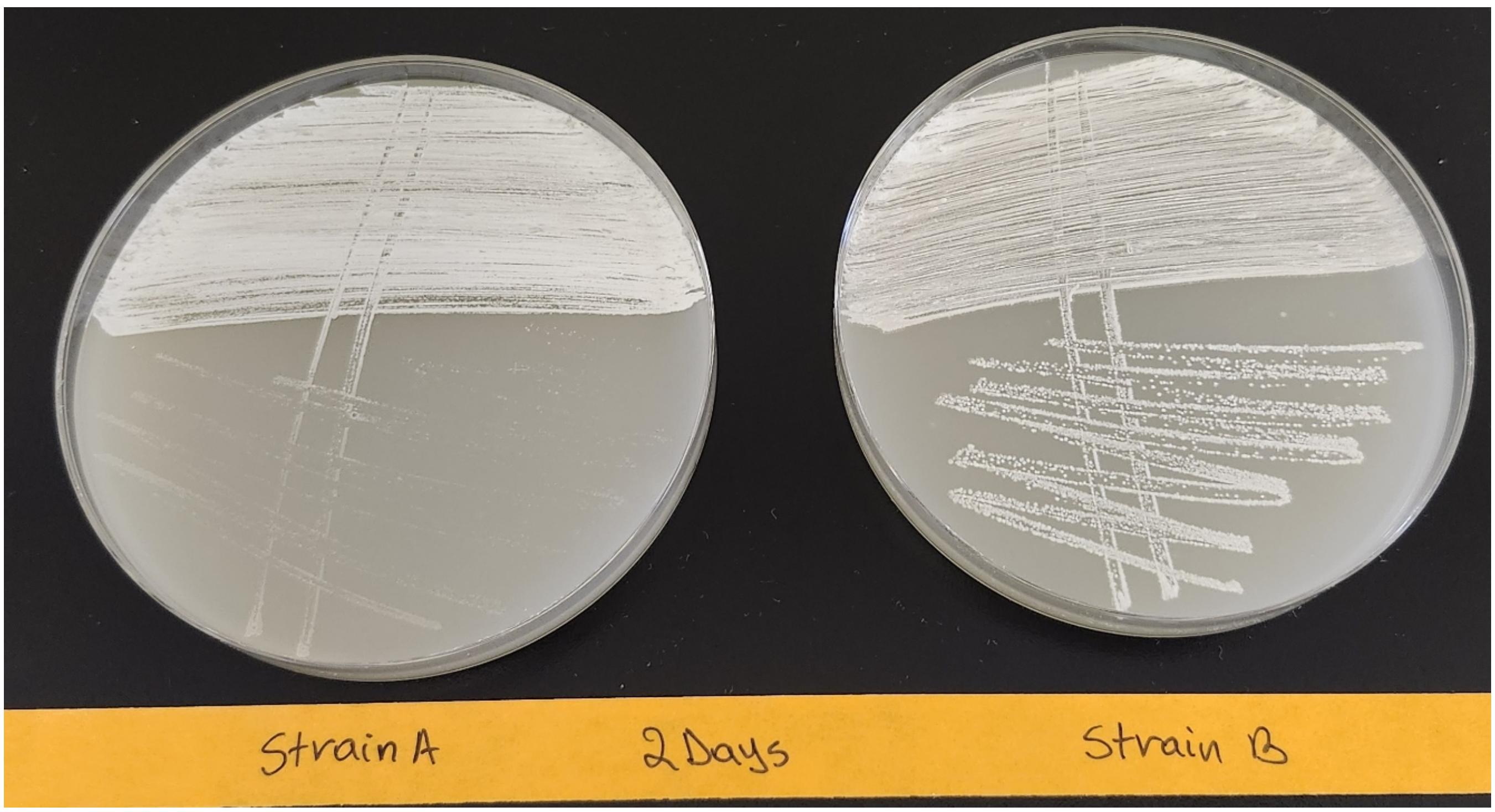

Supplementary Fig. 8. Wild-type Strain A & Strain B two-days growth. *Streptomyces* Strain A & Strain B were streaked on ISP4 medium and allowed to grow at $30\,^{\circ}\mathrm{C}$ for two days. The plates were imaged with a cell phone camera. When streaked for confluence, Strain A and Strain B produce hyphae at the same time. However, diluted streaking show hyphae on the Strain B plate whereas Strain A shows minimal hyphae.

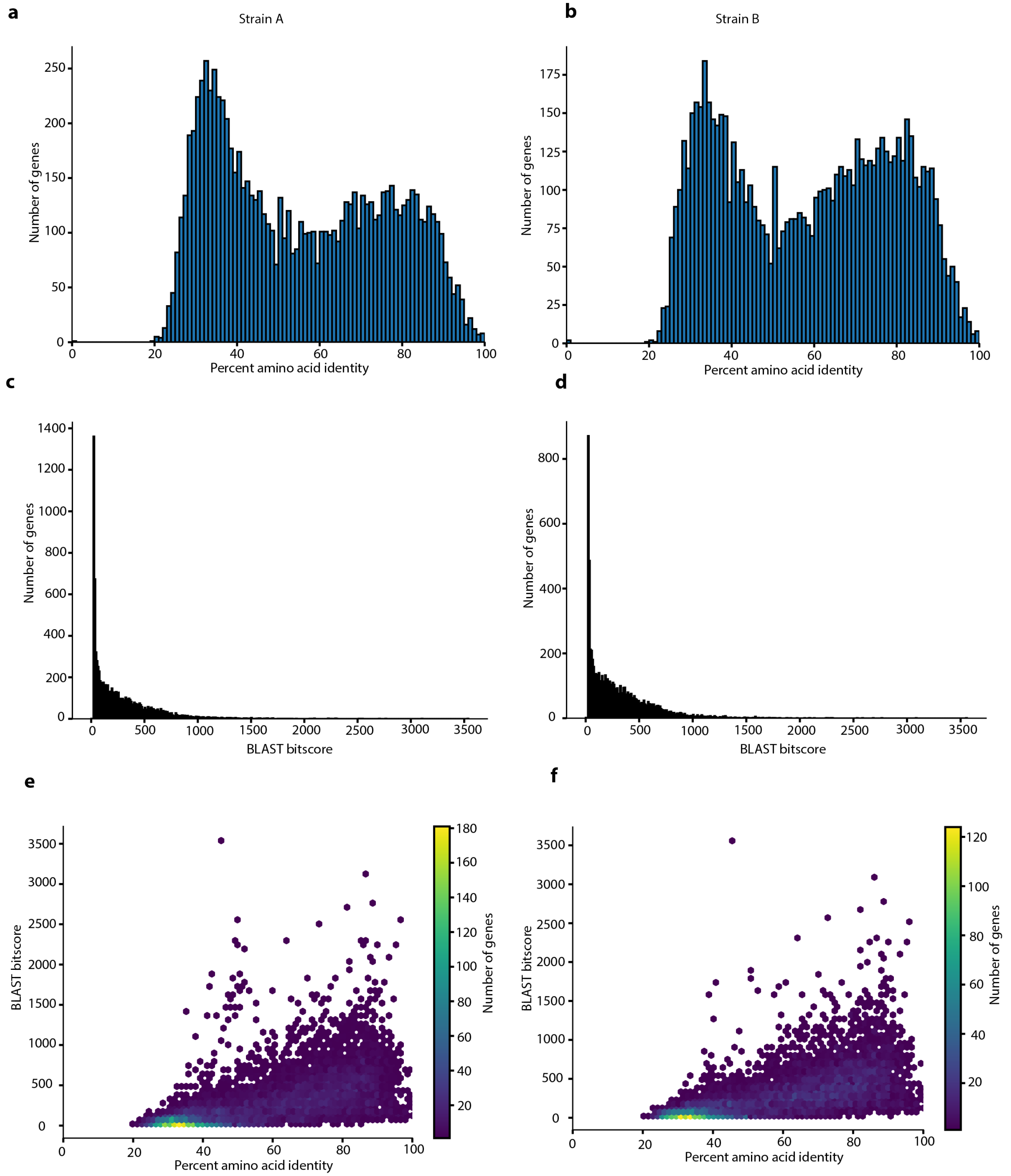

Supplementary Fig. 9. Comparative genomic of Strain A and Strain B. a, c & e, strain A compared to strain B. b, d & f, Strain B compared to Strain A. a & b, Histograms showing the distribution of genes between Strain A and Strain B and vice-versa. The best gene was selected by protein BLAST bitscore. c & d, a histogram of each strain with the protein BLAST bitscore plotted on the x-axis. e & f, Two-dimensional density plot showing gene similarity distribution of Strain A compared to Strain B and Strain B compared to Strain A respectively.

a

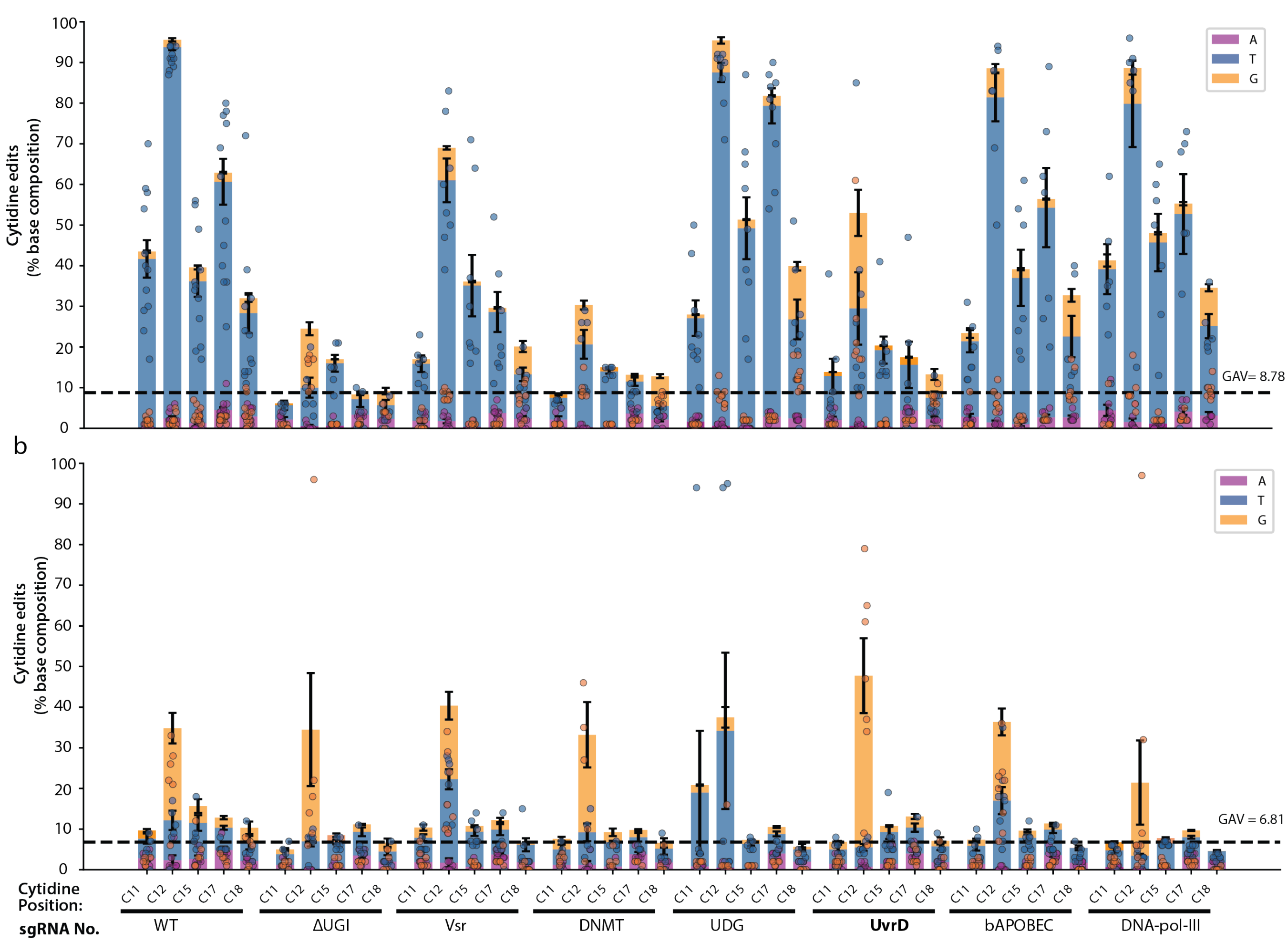

Supplementary Fig. 10. Modified base editing constructs editing in *Streptomyces* Strain A and Strain B. Panels a & b base editing outcomes in Strain A and Strain B with the following constructs: BE3-WT, A-C-ΔUGI, Vsr, DNMT, UDG-like (UDG), UvrD, bAPOBEC, and pol-βsubunit under a constitutive promoter and expressed somewhere else on the base editing plasmid. (a) Base editing results in Strain A. (b) Base editing results in Strain B. Error bar represent the SEM.

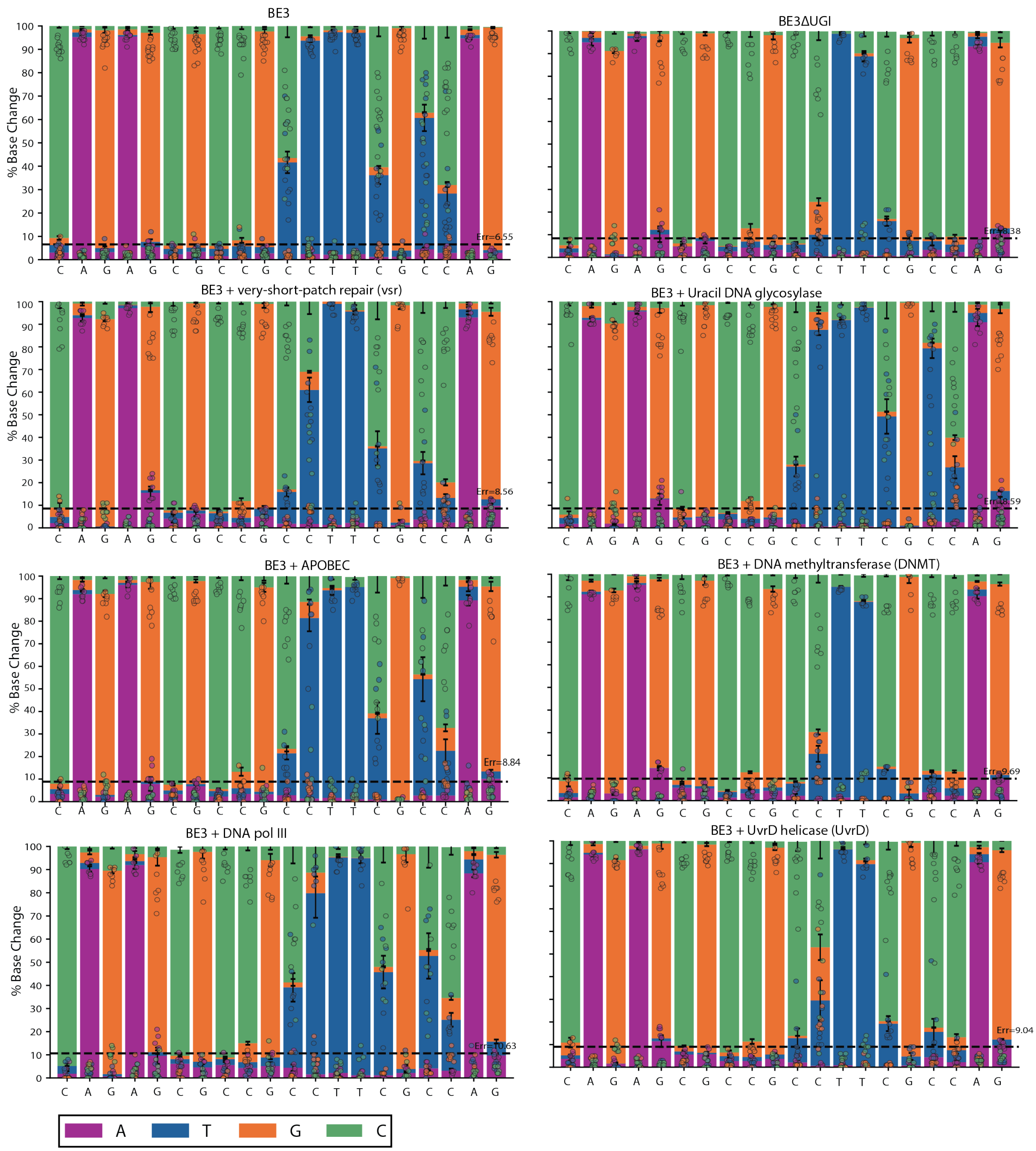

Supplementary Fig. 11. Complete base-editing dataset for Engineered *Streptomyces* Strain A targeting β-glucoronidase. Base-calling frequencies derived from Sanger sequencing chromatograms are shown for each of the nine sgRNA target sites evaluated in *Streptomyces* Strain A for constructs: BE3, BEΔUGI, Vsr, UDG-like, bAPOBEC, DNMT, pol-βsubunit and UvrD. The complete protospacer sequence for each sgRNA is displayed in the $5'$–$3'$ orientation. The dashed line indicates the global average variation (GAV) calculated for each sgRNA target, and error bars represent the standard error of the mean (SEM).

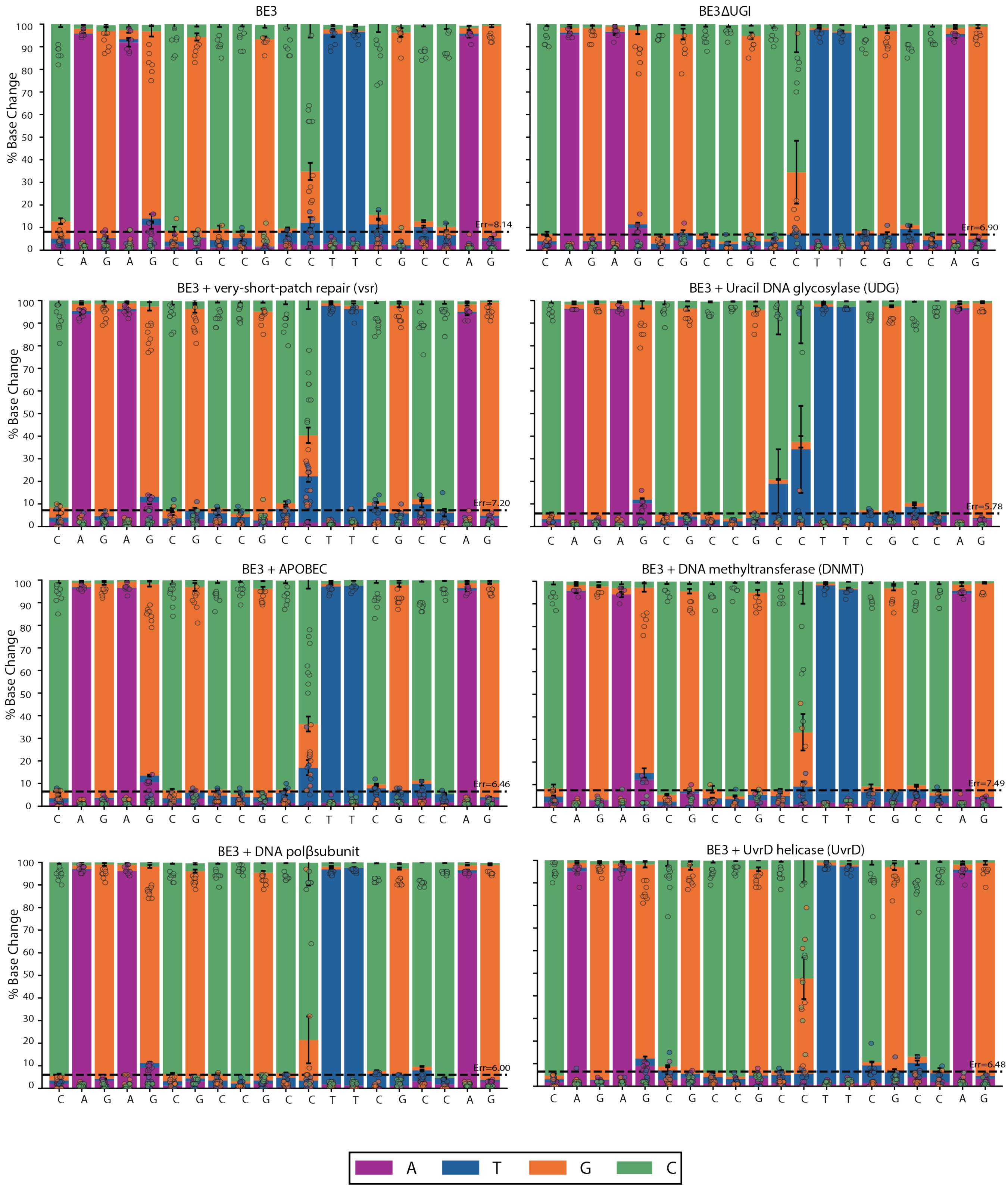

Supplementary Fig. 12. Complete base-editing dataset for Engineered *Streptomyces* Strain B targeting β-glucoronidase. Base-calling frequencies derived from Sanger sequencing chromatograms are shown for each of the nine sgRNA target sites evaluated in *Streptomyces* Strain B for constructs: BE3, BEΔUGI, Vsr, UDG-like, bAPOBEC, DNMT, pol-βsubunit and UvrD. The complete protospacer sequence for each sgRNA is displayed in the 5′–3′ orientation. The dashed line indicates the global average variation (GAV) calculated for each sgRNA target, and error bars represent the standard error of the mean (SEM).

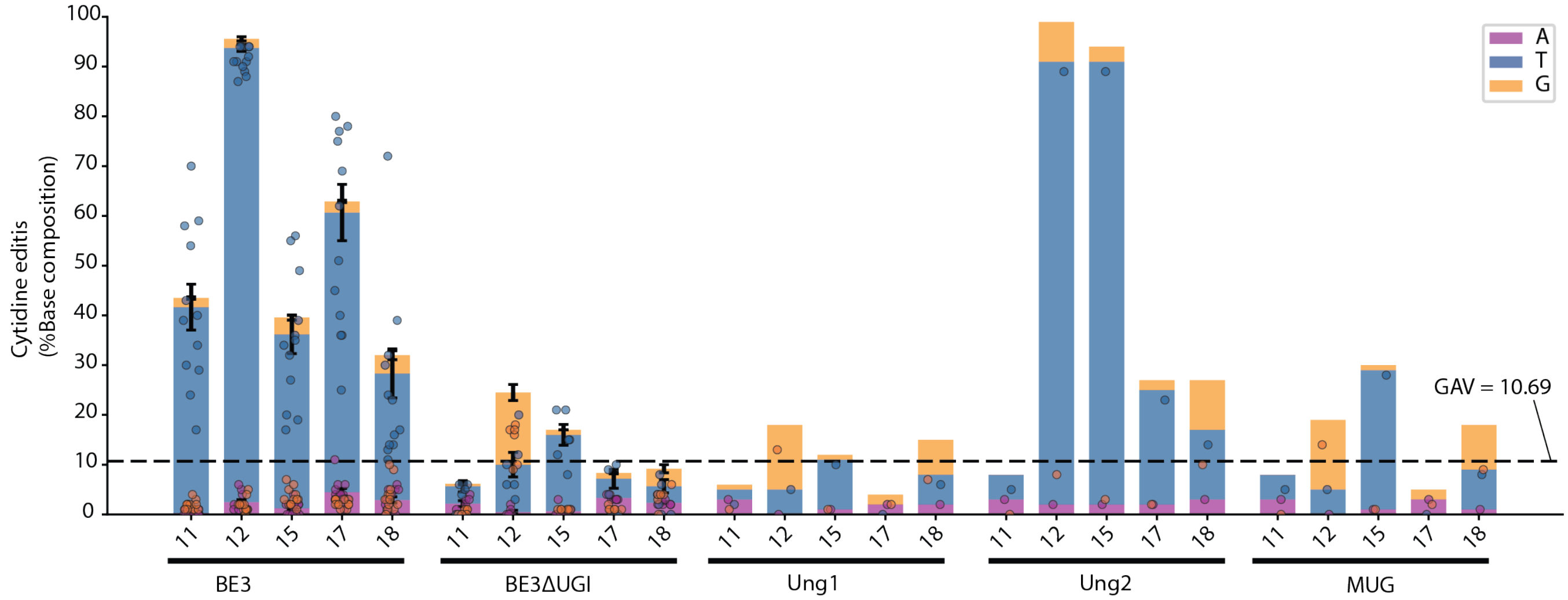

Supplementary Fig. 13. Additional Strain B putative base-excision repair proteins tested in Strain A. Ung1, Ung2 and MUG from Strain B expressed on the base editing plasmid under the constitutive promoter kasOP. Ung1, Ung2 and MUG from Strain B where not flagged in the bioinformatics work as divergent from those in Strain A. The dashed line indicates the global average variation (GAV) calculated for each sgRNA target, and error bars represent the standard error of the mean (SEM).

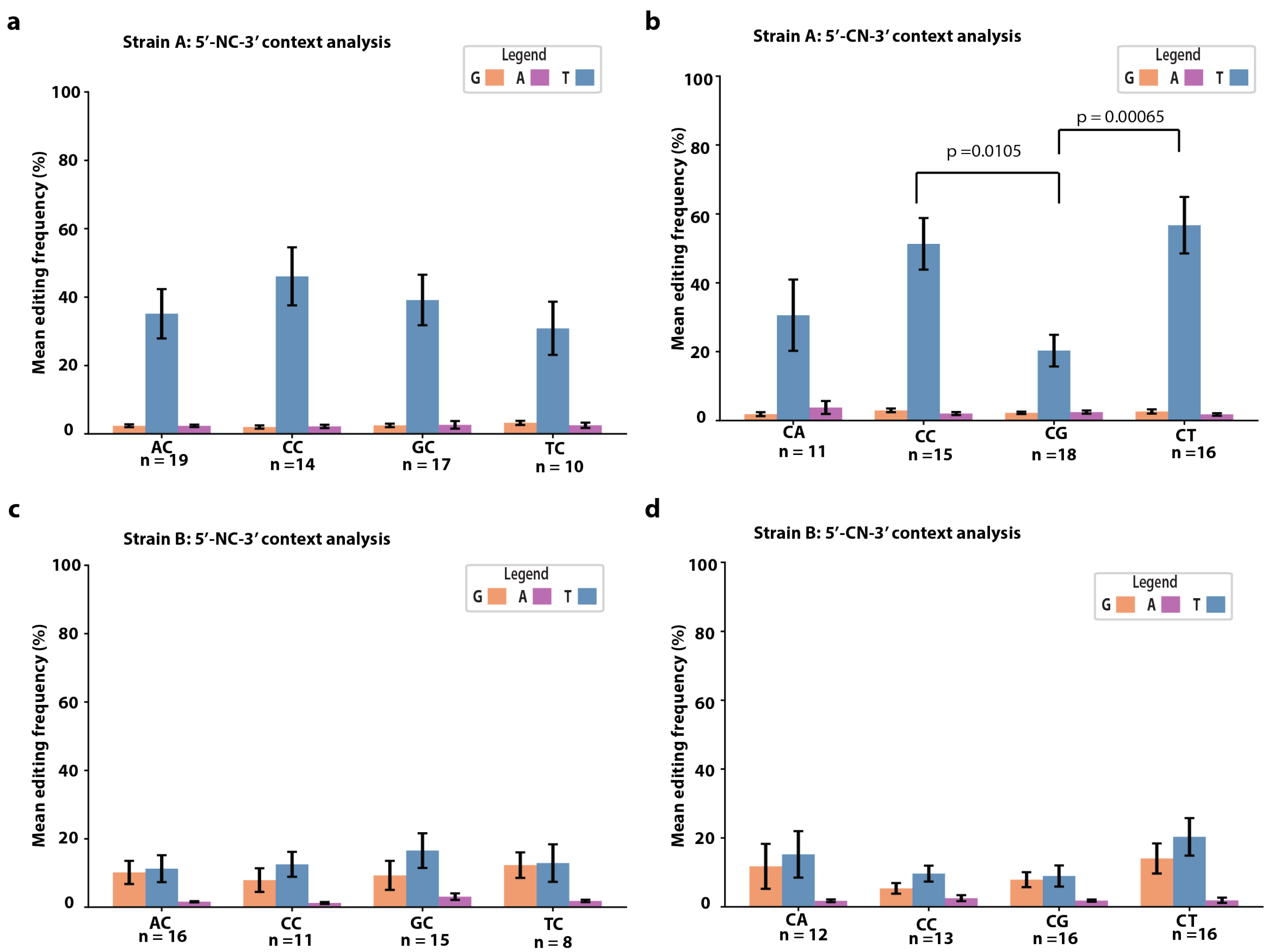

Supplementary Fig. 14. Dinucleotide context influences base-editing outcomes in *Streptomyces* Strain A and Strain B. Panels (a) and (b) show the dinucleotide analysis for *Streptomyces* Strain A, whereas panels (c) and (d) show the corresponding analysis for *Streptomyces* Strain B. Panels (a) and (c) summarize editing frequencies in the 5′–NC–3′ sequence context, where N represents any nucleotide immediately upstream of the edited cytosine. Panels (b) and (d) summarize editing frequencies in the 5′–CN–3′ sequence context, where N represents any nucleotide immediately downstream of the edited cytosine. Statistical significance was assessed using the Kruskal-Wallis test followed by Dunn's post-hoc test with Holm correction. Significant pairwise comparisons are shown above the bars.

Supplementary Table 1. Strains and plasmids used in this study. Putative DNA repair proteins from *Streptomyces* Strain B were added to the base-editing plasmid under the constitutive promoter kasOP. These plasmid constructs were tested in *Streptomyces* Strain A and *Streptomyces* Strain B.

Name	Sequence	Description
**Strains**		
Streptomyces Strain A	N/A	*Streptomyces* strain isolated from disease-suppressive soils.
Streptomyces Strain B	N/A	*Streptomyces* strain isolated from disease-suppressive soils.
**Base-editing plasmids and sgRNA constructs**		
pCRISPR-cBEST	N/A	*Streptomyces*-codon-optimized nCas9 base-editing plasmid (1).
MB383-StrainB-R23-RS31925_V29I	CGGTTGGTAGGATCGACGGGGCGACGAAGACCGAAGCTGGTTTTAGAGCTAGAAATAGC	Targets *Streptomyces* Strain B antiSMASH BGC region 23, gene RS31925, at amino acids V29I
MB477-StrainA-R10-1535_A28T,W30*	cggttggtaggatcgacggctcccagaacgcggtcgcgtcgttttagagctagaaatag	Targets *Streptomyces* Strain A antiSMASH BGC region 10, gene 1535, at amino acids A28T and W30*
MB479-StrainA-R10-1535_P140L,Q141*	cggttggtaggatcgacggccaacccgcagctgtccgactgttttagagctagaaatag	Targets *Streptomyces* Strain A antiSMASH BGC region 10, gene 1535, at amino acids A28T and W30*
MB513-StrainA-R14-2063_W286*	cggttggtaggatcgacggcctgccaggggtgcaggggcagttttagagctagaaatagc	Targets *Streptomyces* Strain A antiSMASH BGC region 14, gene 2063, at amino acid W286.
MB515-StrainA-R14-2067_Q78*	cggttggtaggatcgacggcgcagctcggcgtccagctgcgttttagagctagaaatagc	Targets *Streptomyces* Strain A antiSMASH BGC region 14, gene 2067, at amino acids Q78*.
MB523_StrainA_R14-2091_Q95&94*	cggttggtaggatcgacggcgcagcagggcgggttcctgcgttttagagctagaaatagc	Targets *Streptomyces* Strain A antiSMASH BGC region 14, gene 2091, at amino acids Q95 and Q94.
MB525-StrainA-R14-2081_Q58*	cggttggtaggatcgacggcccagctcatgggtgccctgcgttttagagctagaaatagc	Targets *Streptomyces* Strain A antiSMASH BGC region 14, gene 2081, at amino acids Q58.
MB531-StrainA-R16-2586_Q20*	cggttggtaggatcgacggcccagtcgtacaagccggacggttttagagctagaaatagc	Targets *Streptomyces* Strain A antiSMASH BGC region 16, gene 2586, at amino acids Q58
MB546-StrainA-R20-4464	ggaccagttcgtgggctcct	Targets *Streptomyces* Strain A antiSMASH BGC region 20, gene 4464, at amino acids W85*
MB547-StrainA-R20-4442	cggcagctcgccgacgccgc	Targets *Streptomyces* Strain A antiSMASH BGC region 20, gene 4464, at amino acids Q26 and L27
MB556-StrainB-RS42835_L4	CAAGCTCAACGCGGGCCTGA	Targets *Streptomyces* Strain B genomic locus 7240296-7240315, gene RS42835, at amino acid L4
MB658-StrainB-RS26710	ggtcatgacgcgcagcgtat	Targets *Streptomyces* Strain B genomic locus 5,714,541-5,714,560 gene RS26710, at amino acid M1
MB601-StrainB-dnaA	CATCCAGTTCCTCGCGAGCA	Targets *Streptomyces* Strain B genomic locus 4,220,426-4,220,445 computationally annotated dnaA gene, at amino acid I358, and Q359
MB662-StrainB-RS14720	cgtgccagaagcagccgtcg	Targets *Streptomyces* Strain B genomic locus 3,182,264-3,182,283 gene RS14720, at amino acid W72, and H73
MB663-StrainB-RS14720	caccattcggcgttcgcctt	Targets *Streptomyces* Strain B genomic locus 3,182,310-3,182,329 gene RS14720, at amino acid E87, and W88
MB669-StrainB-RS26710	gcttcagtttccctcgtcga	Targets *Streptomyces* Strain B genomic locus 5,714,816-3,714,835 gene RS26710, at amino acid S89
pMB520_c6_cBEST	N/A	**This work**; pCRISPR-cBEST modified for high-throughput sgRNA insertion using AarI Type IIS cloning.
pMB624_cBEST_ΔUGI-Vsr	N/A	UGI replaced with very-short-patch repair protein Vsr.
pMB625_cBEST_ΔUGI	N/A	UGI removed.

Continued on next page

Supplementary Table 1. Strains and plasmids used in this study, continued.

Name	Sequence	Description
pMB691_cBEST_ΔUGI-Vsr	N/A	UGI removed; Vsr expressed from the constitutive kasOP promoter.
pMB692_cBEST_Vsr_Strain_B	N/A	Vsr expressed from the constitutive kasOP promoter; UGI preserved as a fusion to the nCas9 C-terminus.
pMB710_cBEST_gusA_sgRNA1	caacgaccagttcgcggacg	*gusA*-targeting sgRNA 1.
pMB711_cBEST_gusA_sgRNA2	gacccagaccttgccgtagt	*gusA*-targeting sgRNA 2.
pMB712_cBEST_gusA_sgRNA3	gactggcaggtcgtcgccaa	*gusA*-targeting sgRNA 3.
pMB713_cBEST_gusA_sgRNA4	gagcagcaggtcgtggcgac	*gusA*-targeting sgRNA 4.
pMB714_cBEST_gusA_sgRNA5	cggcaagtcggtccgcatca	*gusA*-targeting sgRNA 5.
pMB715_cBEST_gusA_sgRNA6	cggctacacgcccttcgagg	*gusA*-targeting sgRNA 6.
pMB716_cBEST_gusA_sgRNA7	gttgcagtcctgggccacgt	*gusA*-targeting sgRNA 7.
pMB717_cBEST_gusA_sgRNA8	cctgccagtccacggacgcg	*gusA*-targeting sgRNA 8.
pMB718_cBEST_gusA_sgRNA9	cgatccagtccatcagggcg	*gusA*-targeting sgRNA 9.
pMB719_cBEST_gusA_sgRNA10	ctgctgggtttcgccgttga	*gusA*-targeting sgRNA 10.
pMB720_cBEST_DNMT	N/A	Adds *Streptomyces* DNA methyltransferase to pMB520.
pMB721_cBEST_UDG	N/A	Adds a predicted uracil DNA glycosylase-related protein to pMB520.
pMB742_cBEST_uvrD	N/A	Adds *uvrD* helicase to pMB520.
pMB744_cBEST_cytidine_deaminase	N/A	Adds cytidine deaminase to pMB520.
pMB745_cBEST_DNApol_sliding_clamp	N/A	Adds DNA polymerase sliding clamp to pMB520.
pMB765_cBEST_glycerol_kinase_sgRNA1	CTGGCTGCTCGACAACGTCG	Glycerol kinase sgRNA 1.
pMB766_cBEST_glycerol_kinase_sgRNA2	GACCGCTTCCGCCGCGAGAC	Glycerol kinase sgRNA 2.
pMB767_cBEST_glycerol_kinase_sgRNA3	GGTGAGGTTCCAGATGACCC	Glycerol kinase sgRNA 3.
pMB768_cBEST_glycerol_kinase_sgRNA4	ACCACGGTCGGCTACAAGAT	Glycerol kinase sgRNA 4.
pMB769_cBEST_glycerol_kinase_sgRNA5	GGCGATCACACCGCGGGCGT	Glycerol kinase sgRNA 5.
pMB770_cBEST_glycerol_kinase_sgRNA6	GACGCCCGCGGTGTGATCGC	Glycerol kinase sgRNA 6.
pMB771_cBEST_glycerol_kinase_sgRNA7	GTCGCCGAGACCACCTGCCT	Glycerol kinase sgRNA 7.
pMB799_cBEST_DNA_polβ_subunit	N/A	Adds DNA polymerase β subunit; contains an extra AarI site, so sgRNA insertion requires an additional ligation step.
**Putative endogenous base excision repair constructs**		
pMB811_cBESTΔUGI_Vsr	N/A	UGI replaced with very-short-patch repair protein Vsr from *Streptomyces* Strain B.
pMB812_cBESTΔUGI_Vsr	N/A	UGI removed; Vsr expressed from the constitutive kasOP promoter.
pMB813_cBEST-Vsr	N/A	Preserves BE3 architecture and expresses Vsr from the constitutive kasOP promoter.
pMB814_cBEST-DNMT	N/A	Preserves BE3 architecture and expresses DNA cytosine-5 methyltransferase under the constitutive kasOP promoter.
pMB815_cBEST-UDG	N/A	Preserves BE3 architecture and expresses a predicted uracil DNA glycosylase-related protein under the constitutive kasOP promoter.
pMB816_cBEST-uvrD	N/A	Preserves BE3 architecture and expresses superfamily I DNA/RNA helicase UvrD under the constitutive kasOP promoter.
pMB817_cBEST-ung1	N/A	Expresses *Streptomyces* Strain B uracil DNA glycosylase Ung1 under the kasOP promoter.

Continued on next page

Supplementary Table 1. Strains and plasmids used in this study, continued.

Name	Sequence	Description
pMB818_cBEST-bAPOBEC	N/A	Preserves BE3 architecture and expresses *Streptomyces* Strain B APOBEC under the kasOP promoter.
pMB819_cBEST-DNA_polIII	N/A	Preserves BE3 architecture and expresses DNA polymerase III delta-prime subunit under the kasOP promoter.
pMB828_cBEST_mug	N/A	Preserves BE3 architecture and expresses *Streptomyces* Strain B double-stranded uracil DNA glycosylase Mug under the kasOP promoter.
pMB829_cBEST_ung2	N/A	Expresses *Streptomyces* Strain B uracil DNA glycosylase Ung2 under the kasOP promoter.
pMB830_cBEST_ung1	GACCGCTTCCGCCGCGAGAC	Glycerol kinase sgRNA 2 inserted into pMB817.
pMB831_cBEST_mug	GACCGCTTCCGCCGCGAGAC	Glycerol kinase sgRNA 2 inserted into pMB828.
pMB832_cBEST_ung2	GACCGCTTCCGCCGCGAGAC	Glycerol kinase sgRNA 2 inserted into pMB829.

Supplementary Table 2. Oligonucleotides used in this work. Sequences are written 5'–3'. sgRNAs are shown as 5'–ACGC–FWD–protospacer–3' and 5'–AAAC–REV–protospacer–3'. The asterisk denotes an sgRNA intended to create a premature stop codon at the target site in *Streptomyces* Strain A and *Strain B*.

Name	Sequence (5'–3')
MB964_F_Strain_A&B_gusA_N52	ACGCcaacgaccagttcgcggacg
MB965_R_Strain_A&B_gusA_N52	AAACcgtccgcgaactggtcgttg
MB966_R_Strain_A&B_gusA_W98*	ACGCgacccagaccttgccgtagt
MB967_F_Strain_A&B_gusA_W98*	AAACactacggcaaggtctgggtc
MB968_F_Strain_A&B_gusA_Q205*	ACGCgactggcaggtcgtcgccaa
MB969_R_Strain_A&B_gusA_Q205*	AAACttggcgacgacctgccagtc
MB970_F_Strain_A&B_gusA_Q221,222*	ACGCgagcagcaggtcgtggcgac
MB971_R_Strain_A&B_gusA_Q221,222*	AAACgtcgccacgacctgctgctc
MB972_F_Strain_A&B_gusA_K126	ACGCcggcaagtcggtccgcatca
MB973_R_Strain_A&B_gusA_K126	AAACtgatgcggaccgacttgccg
MB974_F_Strain_A&B_gusA_Y111	ACGCcggctacacgcccttcgagg
MB975_R_Strain_A&B_gusA_Y111	AAACcctcgaagggcgtgtagccg
MB976_R_Strain_A&B_gusA_C197	ACGCgttgcagtcctgggccacgt
MB977_F_Strain_A&B_gusA_C197	AAACacgtggcccaggactgcaac
MB978_R_Strain_A&B_gusA_W204*	ACGCcctgccagtccacggacgcg
MB979_F_Strain_A&B_gusA_W204*	AAACcgcgtccgtggactggcagg
MB980_R_Strain_A&B_gusA_W320*	ACGCcgatccagtccatcagggcg
MB981_F_Strain_A&B_gusA_W320*	AAACcgccctgatggactggatcg
MB982_F_Strain_A&B_gusA_Q385	ACGCctgctgggtttcgccgttga
MB983_R_Strain_A&B_gusA_Q385	AAACtcaacggcgaaacccagcag
MB984_R_Strain_A&B_gusA_screen	gccgacgaccgcgctgacgcggtcgaac
MB985_F_Strain_A&B_gusA_screen	atgctgcggcccgtcgaaaccccgacc
MB991_R_pMB22_amp_traJ_A&B_screen	ctagctagagtcgacctgcaggtccccggggcacgcgcacccccggccgttttagcggc
MB1060_F_Strain_A_attB_screen	cccgtcgatgatgtaggtgacggtctcg
MB1061_R_Strain_A_attB_screen	ccacctcgacccgttcatcatgatggacc
MB1064_F_Strain_B_attB_screen	cctcgacccgttcatcatgatggaccagatgggcgag
MB1065_R_Strain_B_attB_screen	ccgtcgatcaggtacgtgacggtctcgaagcc
MB1074_R_gusA_seq	caggagtcgcgcgccatcgc
MB1075_R_gusA_seq	gcgggaggtgttcatccccaagg
MB1076_R_gusA_seq	gtgagggctacctgtacgagctgtgc
MB1106_F_Strain_A&B_glycerol-sgRNA1	acgcCTGGCTGCTCGACAACGTCG
MB1107_R_Strain_A&B_glycerol-sgRNA1	aaacCGACGTTGTCGAGCAGCCAG
MB1108_F_Strain_A&B_glycerol-sgRNA2	acgcGACCGCTTCCGCCGCGAGAC
MB1109_R_Strain_A&B_glycerol-sgRNA2	aaacGTCTCGCGGCGGAAGCGGTC
MB1110_R_Strain_A&B_glycerol-sgRNA3	acgcGGTGAGGTTCCAGATGACCC
MB1111_F_Strain_A&B_glycerol-sgRNA3	aaacGGGTCATCTGGAACCTCACC
MB1112_F_Strain_A&B_glycerol-sgRNA4	acgcACCACGGTCGGCTACAAGAT
MB1113_R_Strain_A&B_glycerol-sgRNA4	aaacATCTTGTAGCCGACCGTGGT
MB1114_R_Strain_A&B_glycerol-sgRNA5	acgcGGCGATCACACCGCGGGCGT
MB1115_F_Strain_A&B_glycerol-sgRNA5	aaacACGCCCGCGGTGTGATCGCC
MB1116_F_Strain_A&B_glycerol-sgRNA6	acgcGACGCCCGCGGTGTGATCGC
MB1117_R_Strain_A&B_glycerol-sgRNA6	aaacGCGATCACACCGCGGGCGTC
MB1118_F_Strain_A&B_glycerol-sgRNA7	acgcGTCGCCGAGACCACCTGCCT
MB1119_R_Strain_A&B_glycerol-sgRNA7	aaacAGGCAGGTGGTCTCGGCGAC
MB1120_F_Strain_A_glycerol_kinase_amp	GTGACCGACGCCCACACCGC
MB1121_R_Strain_A_glycerol_kinase_amp	CAGTCCTCGTCCTCGATCCAGCCC
MB1122_F_Strain_B_glycerol_kinase_amp	ATGACCACCAGCACCGGCCC
MB1123_R_Strain_B_glycerol_kinase_amp	TCAGCTGGCGTCTTCGTCGTCGAC
MB1124_F_Strain_B_glycerol_kinase_seq	GATCTGGACCAACGTCCAGG
MB1125_F_Strain_B_glycerol_kinase_seq2	ACCGCCGCGGACGTCAAGGCCGTC
MB1126_F_Strain_B_glycerol_kinase_seq3	GCCGGTGTCCCGGTCGCCTC
MB1127_F_Strain_B_glycerol_kinase_seq4	CTACGCGCTGGAGGGCTCCATCG
MB1128_F_Strain_A_glycerol_kinase_seq	GGACGAACGTCCAGGAGGTCGTC

Continued on next page

Supplementary Table 2. **Oligonucleotides used in this work**, continued.

Name	Sequence (5'–3')
MB1129_F_Strain_A_glycerol_kinase_seq2	CGGCGGCATCCCGGTCGCCTCCGC
MB1130_F_Strain_A_glycerol_kinase_seq3	GGGCTCGATCGCCGTCACCGGC
MB1131_F_Strain_A_glycerol_kinase_seq4	CCCGGTACGTCACCAAGGCGCAC
MB1131_F_Strain_A_glycerol_kinase_seq4	CCCGGTACGTCACCAAGGCGCAC
**Assembly oligonucleotides**	
MB956_F_P9R9-vsr_amp_ultramer	TGAGCTGATACCGCTCGCCGCAGCCGAACGACCGAGCGCAGCGAGTCAGT GAGCGAGCAGGTCGGCTGGTTGGCTGACAAAGTGCGTAGGATGACTGTGC ATCAATCAGAGAAGGAGGTACCAAATGACCGGTGCCGTCCCCTCC
MB957_R_Vsr_TerT1_GapdhpEL-amp_ultramer	GCCGACGGCTGCGGTAAGGTGCCCGTAGACGCACGTCCGACCGAAGGAGC AGCGGACCGAAACAAAAAAAGACGCTTTTCAGCGTCTTTAATAATTGGTT TGGTACCGAGCTAGCGTCCCTGCGTGCCGG
MB962_R_cBEST_amp_L4440	CTCGCTCACTGACTCGCTGCGCTCGGTCGTTCGGCTGCGGCGAGCGGTAT CAGCTCACTC

Supplementary Table 3. The amino acid sequences for the *Streptomyces* Strain B divergent putative DNA repair proteins.

Name	Amino Acid sequence
Very-short patch repair	MTGAVPSSPGVSARMSRQASRDTTQELAVRRLLHASGLRYRVNLPVPGIRRR<br>TIDIAFTRARIAVFLDGCFWHGCPEHATRPKANAEWWRIKLEKNMSRDRETT<br>AHLETTGWTVLRFWEHETPEAVARTITLTRAGTQGR
DNA polymerase III delta prime subunit	MAQLFHLRPQEAGDVTEELDEAREMILAVETDESRHIEHEATVVLHRRLHDL<br>VHDDSSANEELQEILIGLLASPSTAYDAPDPRSLALLRLVDDATPPPGVSRR<br>AMGKLISRRSPRDEVLQSVATVPVPVPLGTARMLPQDLPSFTGRDRELRYLL<br>ESGTRTGESPVVLLHGMPGVGKTALAVHAAHALADRFPDGQLFINLRGHAPA<br>DDPAAPLQLLGSLLVSAGIPLSVQPQSVHGRAGLWRSWLATRNVLLILDDAA<br>DLPQVQPLLPGYPGCLTLITSRVRLEVPETTHSLPLDVLSPEEAAALFNRML<br>RQRGVATGSPHAAQVARLCGHLPLALAMVSGLLAAHPTWTVTDLVEDLDHFS<br>RRLISPGTGMRSVGQALGTSLRSLEPSQQTFLRRLSLHPGPDLEKRACSVLT<br>GVTIDRADEYLENLYQRSLLSEVSPGRFRMHDLLVSYLDAEPAEENTDRETA<br>REREQRLQRLVDYYQRTATAAYLMEAHLQPAEELRNSLPDSAPPLNDADQAA<br>DWLAAELDNLAACTQFDADHPDAVARISTVVTAHLLRLGRSAVDTAEHVIDA<br>GRQRAVERRDLRAEALFTHLSGIIAVARDDLPRALAGLGRARTLSAQGGWRL<br>GEARALFTLFIVRRLSGTTDEARDALNQARALFAEAEDQRGLARVVEQISRL<br>ESSEARDLAQLTEQAGQLPAQLSLEDIMEQLEQLALALYLETLAATDQEPAD<br>GRPEYLAAGDRVHSELPARDETPAADAGGQGGSGGLPPEPPVPGEVPDEDPE<br>ERPAGDVTSDPDPSVTVEDLDAIDEQVINFWFTDAAPDDQPLLVDTPYTGCF<br>QVGPDHSENLAAGDRAIPLDIIPPAGLPTQWYVMSRTCKLALAGESRDEEPP<br>TAGEQPEWAVEFDLLVLPHAPSEERLVSVTPTSPGTCRIDVLVMVDGDLYRE<br>LTIEFPAESATPSPATPAQGTPATEAPHSQELAPLPGSRNEAAPPAPASAKS<br>EAGSARAALPARRPPNQRSSSTRVTVRSTQNVLARHTALQVPHDWQQPASTL<br>TLNLMPPQAMWKLEVEGKPGQESHGFVPWQPETDAAQWVSEAQEALESYRQH<br>HAGRYNTITPDDISTRLQRFTANADWAAPHTLATADDQQKWSGVANSPQLRE<br>LAMAGSQLFTALFPREHRFHDLIKNLAPGDRLRIHWKDGGPDHIPWPLLYRG<br>SLPSAGHPIQAKDFFGLHLRTTHIARSAETVRVLSADAARAHLMYWGGTNDD<br>ETMRVALEHIDELNQWKPLVLPQGHKQRKEEISAYLCNPDPISLLYVFSQAY<br>SKNGQVRGLRFGSTVKPDDIVLLSEMGEQPLKDQPLVFMNACETSSADHLYS<br>NMLKNHFVRRGSRAYIGSECKVPAGFAARFAHVFFHFLYYRSHRNQPTAAGE<br>ALAQTRKFFWDQYRSIGGLFYSYVNDDQLFVARPGEVEAMHNPRRTTPLTAA<br>RQR
Superfamily I DNA/RNA Helicase UvrD	MGAGGAVSQRAQDARRQERLLREQWQAARRQALRWDTVGEGEQRVLAQLLVL<br>TARGWRLLVDRQWSGTRTADADMLLVGPGGVFVIHVTAGGGSGDGPGEGRAA<br>GLLGATKAAERAVASLGMSPVAVQPLMVFAGQRRDANLGRIRLLGEHEIGPV<br>LLSQRHRLRVESVRAIADHLERVLPDHAGAAVGLQTTTAPAPASPTEPEGHR<br>PSPSDGLFDLEGLRDAALKGAMQAPIEQWMTFLHPDQVALVRRNWSGPARIS<br>GPAGTGKTVVALHRAAHLARRTAGRILYVTFANNLPRVQSTFLRTMAPAVAD<br>RVDFRSLHSWAQEFLQERGIPVRLHGDKAETAFSLAWKNVGRASRLAELDPA<br>PTYWHEEIDYVIKGRGLTRFEEYATLPRRRRKASLHRAHRQSVWALYEAYEA<br>LRAERGVHDFNDVLSLALAEASRRREQPPYAAVIVDEVQDLTLVGVRLLHAL<br>VGDAPNGLLLVGDGQQTVYPGGFRLTDAGIDIRGDRGQVLRTNYRNSKQILD<br>AALTVVADDAFEDLDGERTPGRRDVDLTYHDGDVVRVTGPTVEVHDRQLLDA<br>LRALPEGVLADTALLCPSMRAIGHYQRLLTQAGLAVCQLEHYDGRAVDAVKL<br>GTYRRAKGLEFKNVFLPQHDAVFANVTPAETAGGTRGGTRGGTETPQTARER<br>EELRRSQLFVAMTRARDLLWLGSVTTGRGSGQDAP
ADP-ribosyltransferase domain-containing protein	MTESSAPPALRVVLTDVNEHVVQAWRAAFADTPGIEIRRGSILDEDVDAWVT<br>PTNSRGRMDGGVDAVIKRHLGSGIQVRVQRAIRDRFAGSMPVGSAVCVPSGA<br>AAPRFLISTPTMVQSSQNVSATLNVALACAAAFQAVHRQNRKAPGSIRSVAL<br>VGMGAQTGRVPARVCANLMWTGYTLFHDHWFEDDDELRATIITQLNGIEKRP<br>VEERIRIVPPKAAAAVTNTNSNSNTEGNSPVTDAHDPLALTELFDGGGEPWL<br>PLLKPVIEAQPDAARFIGKGRSPEVVPVRELTFQALKPHAPHRWKVVVFGQN<br>PYPRPESATGIAMFDNTFNDWTDSQFGRVVSIRCIIKAAAMWKYGIAKKTPI<br>ADVRALLKEQKAVQPPEWFQAMLTQGVLLLNASLTASGDGAMGADRHTAFWR<br>PVAERIVEEILKAKQDADEEDRGVVFAWWGAHARNLKKVVLRLQEKYPDVEV<br>RHIDHANPAAQGDIFCEGDHFAMVNEALASLGSDGIDWLPSKGWNEAAAGTD<br>GAGGGVAARMGAFIESTMNLHQLYLERLTSVKDEGLVLPSITGVFDTPLMDF<br>QDAVAPVAATLSGLAGHIDRSRDFGKRRADETSAGLSADAIAALYLYTCESA<br>FYREINAVLRSPDRTRVTPYLPYLRLLFSAVSGLPAHTQPLWRGVSLDLRAQ<br>YPVGRTVTWWGVSSCTSELNVARSFLGSRGKRTLFEVTPAKAVGIRRFSAFT<br>GEEEYILAPGTQLKVTDVQSERGGLCTVRLTEADATPVVC
Bacterial cytidine deaminase (bAPOBEC)	MTTHPHPVDHELIEAAAHIARTRCRSENHTMAAAARADDGRIVTAVNAYHFT<br>GGPCAELVVIGAAAAQGVHALDTIVAVGDRDRGVVPPCGRCRQVLLDYFPSL<br>RVIVGAGDRVRSVPVADLLPESYVWADHQPADE
DNA methyltransferase	LATVACLETDSDAQAVLRANRPSWWLPANGDVAAAASWLAPEVLGLKRGDLD<br>LIAGGPPCQPFSKAAQWNRAARAGMRDPRAQTIHGMFDLIDAFLPKAVLIEN<br>VSGFLQGKESAVDVVMERLDEINRKNKTDYSLSWQIVDSADYGVPQHRQRAI<br>AIAYREGQAVQLPRPTHLGKPVRAWDALWDLNEDKKPEPSGYWSGLLPSIPE<br>GSNYLHHTARGDGEELFGWRTRYWSFLLKLAKDRPSWTLPASPGPSTGPFHW<br>ENRPLSVREQARLQSFPDAWRFSGVERVQRRVVGNATPPLLAEVLGRSIVAQ<br>LELGDGEEREQLMSRRPTLLRPRRRLIPSQDPVLAVPDVYREHIGTKDAHPG<br>HGRGPSPRMVVEEVA

Bibliography

1. Yaojun Tong, Tilmann Weber, and Sang Yup Lee. Crispr/cas-based genome engineering in natural product discovery. *Natural Product Reports*, 36:1262–1280, 9 2019. ISSN 1460-4752. doi: 10.1039/C8NP00089A. URL https://pubs.rsc.org/en/content/articlelanding/2019/np/c8np00089a.
